# Flexible Ferrite Magnetic Composite Films for Electromagnetic Applications

**DOI:** 10.1021/acsmaterialsau.5c00180

**Published:** 2025-12-12

**Authors:** Jui-Yang Hsu, Chih-Huang Lai, Chia-Chen Li

**Affiliations:** Department of Materials Science and Engineering, 34881 National Tsing Hua University, Hsinchu 30013, Taiwan

**Keywords:** thin-film inductor, flexible magnetic composite film, magnetic alignment, particle dispersion, inductance

## Abstract

Anisotropic magnetic composite films composed of MnZn ferrite powders embedded in an epoxy matrix are investigated for flexible thin-film inductor applications. Commercial MnZn ferrites typically contain α-Fe_2_O_3_ impurities that impair magnetic performance; in this study, these impurities are effectively eliminated by thermal annealing at 600 °C under argon. To improve particle dispersion and processability within the composite, the ferrite is surface-modified with 3-glycidoxypropyltrimethoxysilane and coated with SiO_2_, yielding a more dispersible MZ@SiO_2_. This treatment enables the formation of well-aligned particle chains under an external magnetic field, resulting in pronounced magnetic anisotropy and stable permeability. Incorporating FeNi alloy particles into the MZ@SiO_2_-based films further increases saturation magnetization and permeability, though finite element simulations indicate that the performance of larger FeNi particles is relatively insensitive to orientation. Inductor devices fabricated with the hybrid MZ@SiO_2_–FeNi films exhibit improved inductance and quality factor, underscoring their promise for next-generation high-performance magnetic components with tunable anisotropy.

## Graphical abstract

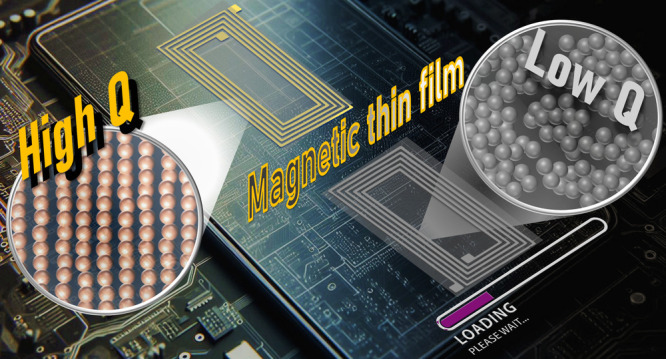

## 1 Introduction

Inductors are indispensable passive components in modern electronic systems, functioning as energy storage elements, filters, and electromagnetic interference (EMI) suppressors across a broad range of applications, including power management modules, radio frequency (RF) circuits, wireless communication systems, and emerging wearable electronics.^1–3^ As devices continue to miniaturize and integrate, the demand for compact, high-performance, and flexible inductors has grown substantially. Flexible magnetic thin films, typically with a thickness of less than 100 μm, have emerged as promising candidates for next-generation flexible inductors due to their lightweight, conformable nature, and process compatibility with roll-to-roll fabrication.^4,5^ Their magnetic properties, including saturation magnetization (Ms) and relative permeability (μ_*r*_), are often tailored by material composition and microstructure.^6^ For instance, soft magnetic materials like Fe-based^7,8^ or Co-based^9^ alloys embedded in a polymer matrix can achieve permeability values around 10 or higher,^10,11^ depending on the loading and dispersion uniformity of magnetic particles. While the inductance values of thin-film inductors are generally in the range from several nanohenries (nH) to microhenries (μH),^12–14^ their quality factor (Q-factor) can vary widely (typically from 5 to 50) at MHz frequencies, influenced by eddy current loss, magnetic loss, and conductor resistance.^15–18^

A key advantage of flexible magnetic thin films lies in their compatibility with microfabrication and patterning techniques such as photolithography, laser ablation, and soft lithography.^19,20^ This enables precise control over device geometry, critical for frequency tuning and integration in compact circuits. Furthermore, patternable films can reduce eddy current pathways, thereby improving high-frequency performance.^21,22^ The performance and mechanical compliance of these films are mainly dependent on the choice of polymer substrates. For instance, polyimide (PI) can offer high thermal stability and mechanical robustness,^23^ making it suitable for device-level integration. Polydimethylsiloxane (PDMS), on the other hand, can provide superior elasticity and optical transparency, which is favorable for wearable and stretchable applications.^24,25^ Epoxy resins are also increasingly employed due to their excellent adhesion, processability, and dimensional stability, and can serve as both structural support and a binder for magnetic particles.^7,26,27^ Each of these polymers brings specific advantages in terms of flexibility, thermal resistance, and chemical compatibility, enabling tailored designs for diverse inductor applications in flexible electronics, RF systems, and power delivery modules.

Importantly, controlling the dispersion of magnetic particles within the polymer matrix is crucial for fabricating high-performance flexible magnetic thin films. Uniform dispersion ensures consistent magnetic performance across the film, enhances mechanical flexibility, and minimizes magnetic loss caused by particle agglomeration. An inhomogeneous distribution of magnetic particles can lead to localized stress concentration, uneven magnetic permeability, and reduced inductance stability, particularly under bending or deformation. Therefore, achieving stable and uniform dispersion is fundamental to the reliability and performance of flexible magnetic devices. However, dispersing magnetic particles into polymer matrices presents significant challenges. Magnetic particles, particularly those with high saturation magnetization (e.g., carbonyl iron,^7,8^ or ferrites^28,29^ ), tend to agglomerate due to strong magnetic dipole–dipole interactions and high surface energy.^30^ This aggregation not only deteriorates the film’s uniformity and magnetic properties but also complicates the coating and patterning processes. To improve compatibility with the polymer matrix and prevent reagglomeration, surface modification of magnetic particles using coupling agents, surfactants, or functionalized polymers is often employed.^7,31^

Among various filler–matrix systems, MnZn ferrite particles embedded in an epoxy matrix offer strong potential for flexible magnetic films used in inductors and EMI suppression.^32,33^ MnZn ferrites offer high initial permeability, low core losses below several MHz, and moderate saturation magnetization, well-suited for low- to midfrequency applications.^34,35^ Their low eddy current losses ensure thermal and magnetic stability, essential for thin-film inductors. Epoxy resins, in turn, provide good mechanical strength, electrical insulation, and strong substrate adhesion, which helps maintain film integrity and particle alignment during curing. Also, epoxy is compatible with scalable processes such as photolithography or screen printing, which is beneficial for patterned or miniaturized devices. In this study, the MnZn ferrite/epoxy system will be investigated with a focus on addressing the dispersion and adhesion challenges encountered during the fabrication of flexible magnetic films. Enhancing the dispersion of MnZn ferrite particles within the epoxy matrix is crucial, as it enables a higher powder content to be achieved, which is directly linked to improved magnetic performance.

## 2 Materials and Methods

### 2.1 Raw Materials

Magnetic powders, including commercially supplied MnZn ferrite (Mn_0.6_Zn_0.4_Fe_2_O_4_; purity >99.9%, CSC Group Himag Magnetic Corporation, Taiwan) and a FeNi alloy with a nominal Fe_50_Ni_50_ composition (purity >99.5%, CSC Group Himag Magnetic Corporation, Taiwan), were used as fillers in the magnetic composite films. The MnZn ferrite powder has an average particle size of approximately 1–2 μm, while the FeNi alloy powder exhibits a larger size of about 30–50 μm. A commercial epoxy (CHENG YI Chemical Co., Ltd., Taiwan) was used as the polymer matrix for the composite films. To improve the compatibility of MnZn ferrite and FeNi alloy powders in the epoxy matrix, a coupling agent (3-glycidyloxypropyl)­trimethoxysilane (GPTMS) (KH560, Echo Chemical Co., Ltd., Taiwan) was employed. The solvent used for mixing the magnetic powders, epoxy, and coupling agent is acetone (Echo Chemical Co., Ltd., Taiwan).

### 2.2 Surface Modification and Annealing Thermal Treatment

To coat the MnZn ferrite particles with SiO_2_, the Stöber method was adopted.^36,37^ The ferrite powder was mixed with tetraethoxysilane (TEOS; Showa Chemical Industry Co., Ltd., Japan), 3-aminopropyltriethoxysilane (APTES; Thermo Fisher Scientific Inc., USA), ethanol, ammonia solution, and deionized water in a mass ratio of 1.86:0.46:0.09:83.64:4.65:9.29. The mixture was stirred at 500 rpm for 6 h, during which the APTES was hydrolyzed to form an amorphous silica layer on the ferrite particle surface. After several washes with ethanol and water, followed by drying at 80 °C for 24 h, the powder was then annealed at 400 °C for 1 h in Ar to remove any residual organic material. The final product is labeled as MZ@SiO_2_. For the FeNi alloy powder, it was annealed at 400, 600, 800, or 1000 °C with a heating rate of 5 °C min^–1^ inside a furnace (STF54434C, Lindberg/Blue M, Thermo Fisher Scientific Inc., USA) filled with high-purity Ar­(g) or *O*_2_(*g*).

### 2.3 Fabrications of Magnetic Composite Films and Planar Inductors

During the film preparation process, the coupling agent GPTMS (5 wt % based on the powder mass) was dissolved in acetone, followed by the addition of a specific amount of magnetic powder (ranging from 10 vol % to 50 vol %, based on the mass of the epoxy added later). This powder suspension was deagglomerated using a high-power ultrasonication horn (Q125, QSONICA, USA) at an output energy of 75 W. Next, the suspension was mixed with epoxy and heated to 70 °C to evaporate most of the acetone. It was then combined with a hardening agent (25% based on the epoxy mass). The resulting magnetic slurry was tape-cast onto an aluminum film, with or without the application of an external magnetic field of 0.04 T to produce an anisotropic magnetic film. After drying and curing at room temperature, the films had an average thickness of 100 μm.

In preparing an air-core planar inductor, the pattern was designed and then printed onto printed-circuit-board (PCB) heat transfer paper. Using a heating and compressing method, the inductor pattern was thermally transferred onto a copper-clad laminate. Subsequently, a sodium persulfate (Na_2_S_2_O_8_) etching solution was used to dissolve excess copper in the nonpatterned areas. After brushing and cleaning, the remaining unetched regions resulted in a PCB with the inductor pattern featuring a line width of 200 μm.^38^ For comparison, another inductor specimen was created by tape casting the magnetic composite slurry onto the top surface of the fabricated planar inductor, forming a homogeneous and even coverage with a layer thickness of 100 μm.

### 2.4 Characterizations and Simulations

To examine the microstructure of the composite films, a scanning electron microscope (SEM; Hitachi, Japan) with an energy-dispersive spectrometer (Horiba, Japan) was used. Surface chemical composition was examined using X-ray photoelectron spectroscopy (XPS; ULVAC-PHI Inc., Japan), while crystal structure analysis was carried out via X-ray diffraction (XRD; Bruker, Germany). Rheological behavior was assessed using a rheometer (Anton Paar, Austria) equipped with a cone–plate configuration (25 mm diameter, 1° cone angle, 0.048 mm gap).^7,39^ The viscoelastic properties of the formulated magnetic slurries were evaluated by recording the storage modulus (G’) and loss modulus (G”) across varying frequencies. Magnetorheological behavior was investigated using a custom device capable of applying a magnetic field with a 20 mm diameter and a 0.1 mm gap. Magnetic properties, including magnetization, were measured using a vibrating sample magnetometer (KLA, USA). The inductance and quality factor of the planar inductor were characterized as a function of frequency using an LCR meter (E4980A, Keysight Technologies, Inc., USA). Relative permeability measurements were performed using a vector network analyzer (PMF-3000, Ryowa Electronics Co., Ltd., Japan). For numerical simulations, finite element analysis (FEA) was conducted in COMSOL Multiphysics (version 5.6) using the AC/DC module, specifically the “magnetic fields, no currents” interface.

## 3 Results and Discussion

### 3.1 Magnetic Properties of MnZn Ferrite

The MnZn ferrite powder used for fabricating the magnetic composite film and planar inductor appears black, exhibits an irregular shape, and has an average particle size of 1–2 μm, as shown in Figure 1a. Based on the M-H loop analysis in Figure 1b (gray line), this commercial ferrite powder demonstrates a notably low magnetization, reaching values below 5 emu cm^–3^ under the applied magnetic fields. The magnetization curve also indicates difficulty in achieving saturation. Using eq 1, the hysteresis M-H loop can be further utilized to calculate the relative permeability (μ_*r*_) of the material (dimensionless):

$$\mu_{r}=4\pi \frac{dM}{dH}+1$$ 1

where μ_*r*_ represents the ratio of the material’s absolute permeability (μ) to that of free space (μ_0_), i.e., μ_*r*_ = μ/μ_0_, with μ_0_ = 4π × 10^–7^ H m^–1^ denoting the permeability of free space. In eq 1, H is the magnetic field intensity, and M is the magnetization of the material (in the same unit as H). Because μ_0_ is a constant, all experimentally determined or calculated values of μ_*r*_ in this work are dimensionless and directly represent the intrinsic magnetic response of the material. The calculated result, shown in Figure 1c (gray line), indicates that the μ_*r*_ of the ferrite powder is nearly equal to one. Such low μ_*r*_ and weak magnetization are common in commercial MnZn ferrite powders and can result from several factors. Deviations from the ideal Mn:Zn:Fe stoichiometry, the presence of impurities, and secondary nonmagnetic phases are known to reduce the magnetic contribution of the ferrite. To clarify the phase composition, XRD analysis was performed, as shown in Figure 1d, which reveals a significant presence of weakly magnetic α-Fe_2_O_3_ in the powder.^40,41^ In addition to the negative impact of α-Fe_2_O_3_, very fine crystallite or grain sizes may cause spin disorder at grain boundaries, further lowering the net magnetization.^42^ Considering the grain size effect, the crystallite size of the MnZn ferrite was estimated using the full width at half-maximum (fwhm) of the (311) diffraction peak at 2θ = 35.1° (Figure 1d), and calculated using the Scherrer eq (eq 2):

$$D=\frac{K\lambda }{\beta \cos\theta }$$ 2

where *D* is the average crystallite size, *K* is the Scherrer constant (typically ∼ 0.9), λ is the X-ray wavelength, β is the fwhm in radians, and θ is the Bragg angle. The calculated crystallite size of the as-received MnZn ferrite was found to be 27.1 nm.

**Figure 1:**
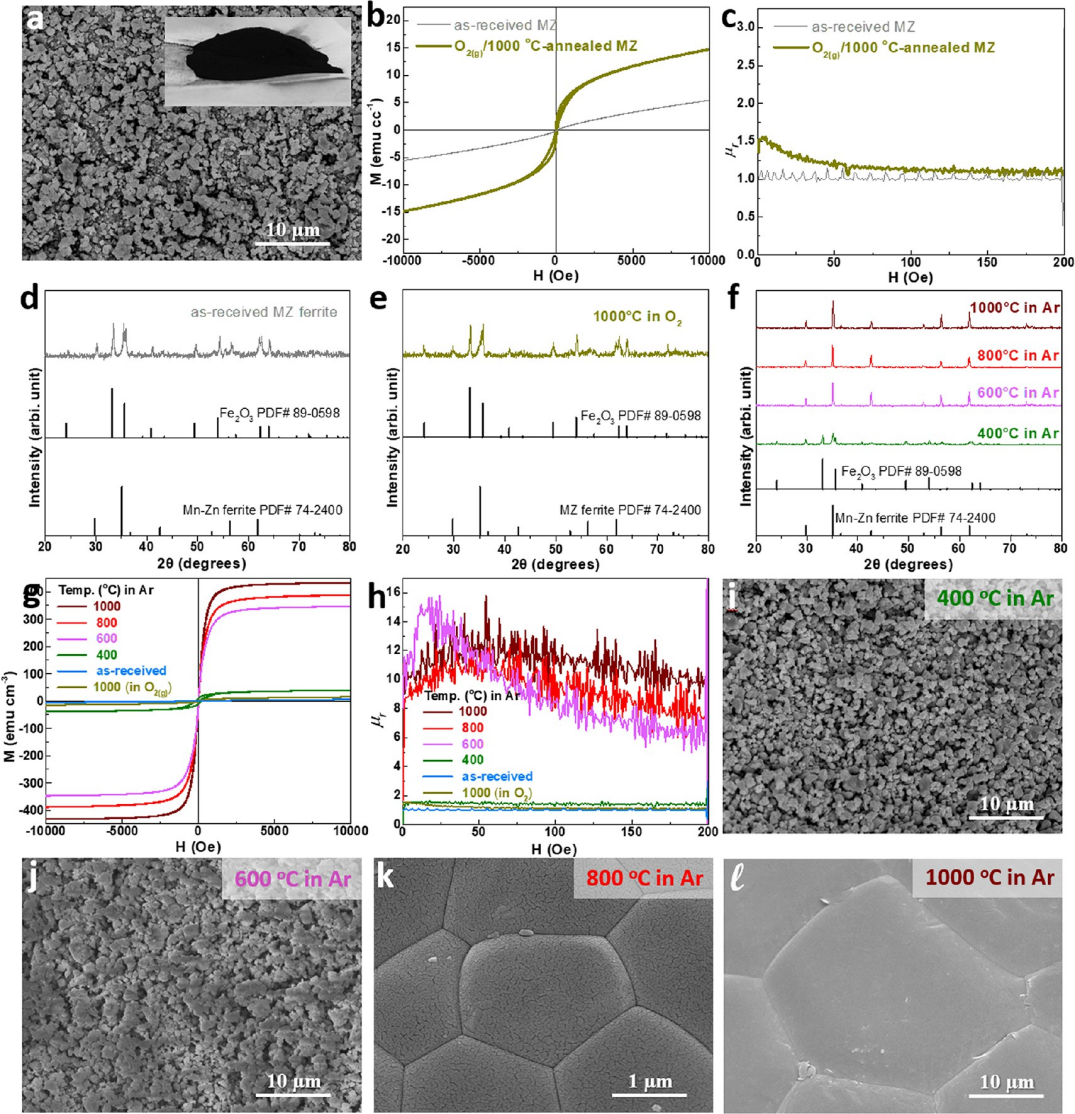
(a, i–l) SEM images, (b, g) M-H loops, (c, h) μ_*r*_ as a function of magnetic field strength, and (d–f) XRD patterns of (d) the as-received MnZn ferrite powder and the powders after annealing (e) under O_2_ at 1000 °C or (f) under Ar at 400–1000 °C.

To increase the crystallite size of the MnZn ferrite powder, thermal annealing was first carried out at 1000 °C under an O_2_ atmosphere. However, as shown in Figure 1b and 1c (dark-yellow lines), only a slight increase in magnetization and μ_*r*_ was observed. To clarify the underlying cause, XRD analysis was performed (Figure 1e), revealing that the presence of α-Fe_2_O_3_ remained significant after the O_2_ annealing treatment. Using eq 2, the crystallite size after annealing at 1000 °C in O_2_ was calculated to be 28.6 nm, indicating only a minor increase compared to the pristine powder. This limited grain growth corresponds well with the minimal improvement in magnetic properties seen in Figure 1b and 1c.

To achieve a more pronounced enhancement in phase purity and magnetic performance, the MnZn ferrite powder was annealed under an Ar atmosphere at various temperatures (400–1000 °C). As shown in Figure 1f, the diffraction peaks associated with α-Fe_2_O_3_, typically originating from incomplete solid-state reactions or surface oxidation during commercial synthesis, disappeared completely after annealing above 600 °C. This transformation can be attributed to the oxygen-deficient environment under Ar, which reduces Fe^3+^ in α-Fe_2_O_3_ and promotes its solid-state reaction with Mn^2+^ and Zn^2+^ to form a homogeneous spinel-type ferrite phase through cation diffusion and redistribution. Similar α-Fe_2_O_3_-to-spinel conversion behavior under low-oxygen annealing conditions has been reported in other ferrite systems, where cation migration and Fe^3+^/Fe^2+^ reordering enable impurity dissolution into the spinel lattice.^34,40,43^ The resulting powders exhibit well-defined spinel structures with crystallite sizes of 51.3 nm at 600 °C and 54.8 nm at 1000 °C (Table 1), confirming effective phase homogenization. In contrast, annealing at 400 °C produced no significant change in crystallite size, indicating insufficient thermal activation for phase conversion. Magnetic characterization (Figure 1g) revealed that samples annealed at 600–1000 °C exhibit markedly increased values of Ms, while the 400 °C sample remains below 50 emu cm^–3^, consistent with the persistence of α-Fe_2_O_3_ impurities. The corresponding value of μ_*r*_ derived from M-H loops (Figure 1h) also increases significantly, exceeding 10 for samples annealed above 600 °C. These findings indicate that annealing MnZn ferrite at temperatures above 600 °C in an inert atmosphere effectively removes α-Fe_2_O_3_ impurities through diffusion-driven phase reconstruction, thereby improving both the crystallinity and magnetic properties essential for magnetic composite applications.

**Table 1:** Crystallite Size of MnZn Ferrite Powders Treated Under Different Thermal Treatment Conditions

treatment of powder	crystallite size (nm)
none (as-received MnZn ferrite)	27.1
1000 °C in O_2(g)_	28.6
1000 °C in Ar_(g)_	54.8
800 °C in Ar_(g)_	53.1
600 °C in Ar_(g)_	51.3
400 °C in Ar_(g)_	31.2

In addition to magnetic properties, Figure 1i-1l compares the microstructures of MnZn ferrite powders subjected to different annealing temperatures. The samples annealed at 400 °C (Figure 1i) and 600 °C (Figure 1j) exhibit average particle sizes comparable to that of the as-received MnZn ferrite. In contrast, notable particle coarsening and densification are observed in the samples annealed at 800 °C (Figure 1k) and 1000 °C (Figure 1l). However, these coarsened particles are difficult to regrind into finer sizes (Figure S1, Supporting Information). Although the powders annealed at 800 and 1000 °C show enhanced Ms and μ_*r*_, their larger particle sizes may hinder thickness control during the fabrication of composite films, thereby limiting their practical utility. Considering both improved magnetic properties and better processability, the MnZn ferrite powder annealed at 600 °C, which also demonstrates superior Ms and μ_*r*_, was selected for subsequent fabrication of magnetic composite films and planar inductors.

### 3.2 Dispersion and Adhesion of MnZn Ferrite in Composite Films

To prepare magnetic composite films using MnZn ferrite, a magnetic slurry was formulated by mixing 40 vol % of the 600 °C-annealed ferrite powder with an epoxy polymer matrix, followed by tape casting. As shown in Figure 2a, the slurry exhibited a dry-out effect caused by inadequate powder dispersion, where particle agglomeration trapped solvent and produced a nonflowable, rough film. Figure 2b presents the rheological behavior, viscosity (η) as a function of shear rate, of ferrite/epoxy slurries with ferrite contents ranging from 10 to 40 vol %. All slurries display shear-thinning behavior, where viscosity decreases with increasing shear rate and eventually levels off at a lower value, indicating the presence of particle agglomerates that are disrupted under shear.^7,37,39,44^ As ferrite content increases, the shear-thinning effect becomes more pronounced, suggesting more severe powder agglomeration. Notably, at 50 vol %, the slurry became too thick to process and prepare successfully.

**Figure 2:**
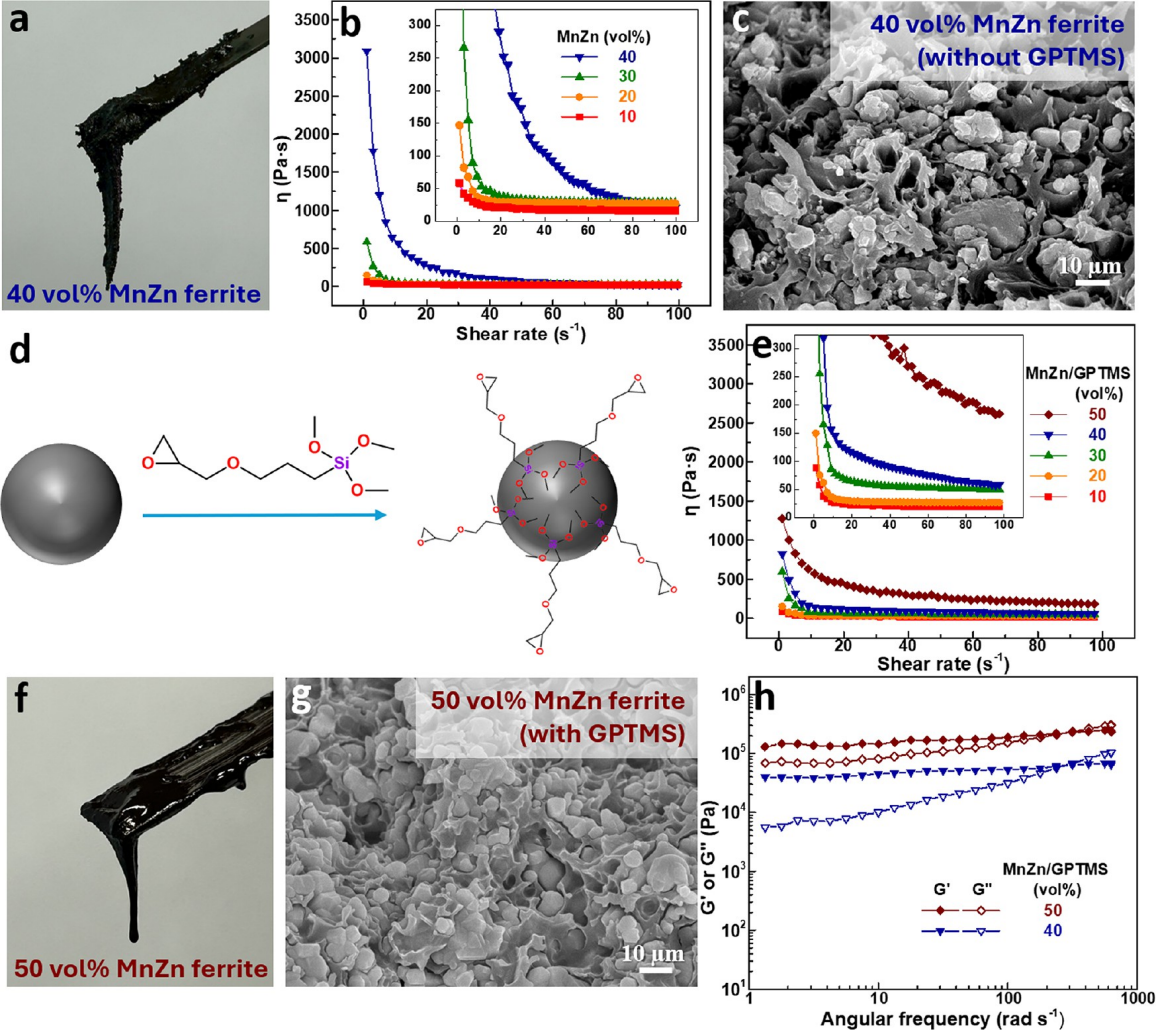
(a, f) Photographs, (b, e) steady-state rheology (viscosity vs shear rate), (h) dynamic rheology (G′ and G″ vs oscillation frequency), and (c, g) cross-sectional SEM images of dried films containing MnZn ferrite (a–c) without and (e–h) with GPTMS (5 wt % relative to ferrite mass). (d) Schematic illustrating the chemical structure of GPTMS and its role in modifying the ferrite particles.

After drying the slurry containing 40 vol % ferrite in epoxy, the cross-sectional image of the dried tape reveals an uneven distribution of ferrite and epoxy, as shown in Figure 2c, along with evident ferrite agglomeration and weak adhesion between the particles and the epoxy matrix (Figure S2, Supporting Information). This poor adhesion, along with the presence of air gaps between ferrite particles and the epoxy, can be detrimental to both the mechanical integrity and magnetic performance of the resulting composite film. To address this issue, the coupling agent GPTMS was introduced. As illustrated in Figure 2d, GPTMS contains silane groups at one end, an epoxide group at the other, and an ether linkage in the middle. With the addition of GPTMS, the viscosity of all ferrite/epoxy slurries decreases significantly, as shown in Figure 2e. Notably, a 50 vol % slurry becomes feasible to process and exhibits even lower viscosity than the 40 vol % slurry without GPTMS, as indicated by the enhanced flowability seen in Figure 2f compared to Figure 2a. After drying, the cross-sectional image of the GPTMS-containing composite film (Figure 2g) shows a clear reduction in ferrite agglomeration, indicating that GPTMS effectively improves ferrite dispersion within the epoxy matrix. This may occur because GPTMS bonds to hydroxylated ferrite surfaces through silane groups and reacts with the epoxy via its epoxide moiety,^45^ thereby improving filler–matrix compatibility and reducing agglomeration.

Additionally, the viscoelastic properties of ferrite/GPTMS/epoxy slurries with powder contents of 40 and 50 vol % were further examined using dynamic rheology, as shown in Figure 2h. Both slurries exhibit notably high values of storage (G’) and loss (G”) moduli, with G’ consistently exceeding G” across most of the measured frequency range. This indicates a pronounced solid-like, elastic behavior,^7^ even though their viscosities are significantly reduced compared to slurries without GPTMS. The strong elastic response and very high values of G’ and G” suggest that rearranging ferrite particles within the epoxy matrix will be difficult, which may hinder effective magnetization and particle alignment during the application of an external magnetic field. Such solid-like behavior can usually be reduced by deagglomerating the particles. For the persistently high G’, it is suggested that GPTMS should not fully adsorb onto the ferrite particle surfaces to achieve complete coverage. This is likely due to the inherently strong magnetic attraction between ferrite particles, which promotes agglomeration. While ferrites, like most oxides, possess surface hydroxyl groups that can react with silane-based coupling agents, the quantity of accessible hydroxyl groups may be insufficient for GPTMS to achieve effective adsorption and coverage. To increase the density of hydroxyl groups and simultaneously reduce particle attraction, premodifying MnZn ferrite particles with a SiO_2_ layer could be an effective approach.

### 3.3 Silica Coating to Enhance MnZn Ferrite Dispersion

Figure 3a shows the MnZn ferrite particles surface-modified with a SiO_2_ layer (denoted MZ@SiO_2_). Elemental mappings in Figure 3b-e confirm the uniform Si distribution along the particle surfaces. Complementary XPS analysis (Figures S3 and S4, Supporting Information) further verifies the surface composition and oxidation states. As shown in Figure 3f, the Fe 2p XPS spectrum of MnZn ferrite annealed under Ar at 600 °C exhibits two dominant peaks at ∼ 711.1 eV and ∼ 725.0 eV, corresponding to the Fe 2p3/2 and Fe 2p1/2 components typical of Fe^3+^ species in spinel ferrites.^7,46^ Satellite peaks near 719.8 and 733.8 eV further corroborate the presence of Fe^3+^. A weak shoulder around 709 eV and a minor satellite near 715 eV (Figure S4, Supporting Information) indicate the existence of a small Fe^2+^ fraction, which can be attributed to localized oxygen deficiency or partial reduction during annealing in an inert Ar environment. Inclusion of this Fe^2+^ component in the deconvolution provides a more accurate fit, suggesting a mixed-valence state within the ferrite lattice. For MZ@SiO_2_, both Fe 2p peaks and their satellites are strongly attenuated due to the overlying SiO_2_ shell, confirming successful coating while preserving the Fe^3+^-dominated oxidation state beneath the surface.

**Figure 3:**
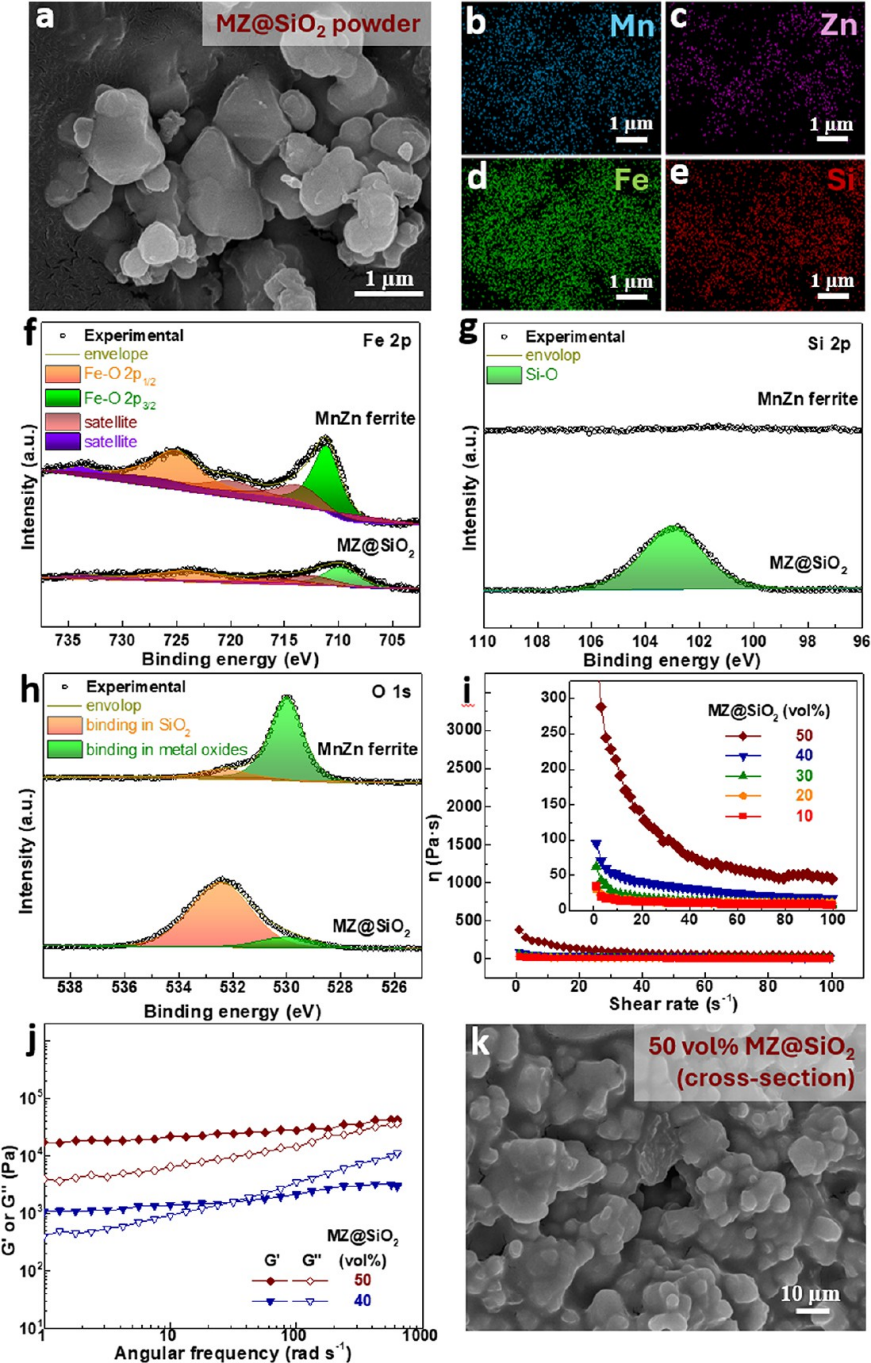
(a) SEM image of MZ@SiO_2_ and corresponding EDS elemental mappings of (b) Mn, (c) Zn, (d) Fe, and (e) Si. (f-h) XPS spectra showing the chemical states of (f) Fe 2p, (g) Si 2p, and (h) O 1s for MnZn ferrite and MZ@SiO_2_. (i) Steady-state rheology (viscosity vs shear rate), (j) dynamic rheology (G′ and G″ vs oscillation frequency), and (k) cross-sectional SEM image of the MZ@SiO_2_/GPTMS/epoxy film.

The Si 2p spectra for the MnZn ferrite and MZ@SiO_2_ are shown in Figure 3g. In the uncoated MnZn ferrite, no distinct Si 2p signal is observed, confirming the absence of silicon species on the particle surface. In contrast, the SiO_2_-coated MZ@SiO_2_ exhibits a strong Si 2p peak centered around ∼ 102.5 eV, which is characteristic of Si^4+^ in silicon dioxide.^47^ For the O 1s spectra in Figure 3h, the MnZn ferrite shows a dominant peak for binding in metal oxides and a smaller peak for binding in SiO_2_. Nevertheless, we have confirmed the absence of silicon species on the MnZn ferrite particle surface, then the small peak at approximately 532.5 eV is more likely attributed to an impurity or contaminant. In contrast, the bottom spectrum for the MZ@SiO_2_ is dominated by a large peak for binding in SiO_2_ and a much smaller peak for binding in metal oxides, confirming again that the surface is almost completely covered by a SiO_2_ shell.

To gain more understanding of the SiO_2_ layer thickness on MZ@SiO_2_, XPS depth profiling was conducted (Figure S3b, Supporting Information). The result shows that with increasing sputter time, the atomic concentration of Si decreases, while the concentrations of other metal elements rise. This indicates that SiO_2_ is more concentrated on the particle surface, forming a shell around 50 nm thick. This value agrees well with the TEM observation (Figure S5, Supporting Information), which shows that the SiO_2_ coating varies in thickness from <10 nm to approximately 60 nm. This nonuniformity likely originates from the irregular morphology and curvature-dependent surface energy of the MnZn ferrite particles, influencing local precursor adsorption during the sol–gel process. Together, the XPS and TEM results confirm the formation of a continuous SiO_2_ shell that effectively modifies the ferrite particle surface.

When the as-synthesized MZ@SiO_2_ powder was mixed with GPTMS and epoxy, the resulting slurry exhibited reduced viscosity and less pronounced shear thinning across a solid content range of 10–50 vol % (Figure 3i), compared to the MnZn/GPTMS/epoxy slurries (Figure 2e). These changes suggest that agglomerates were effectively broken up due to the SiO_2_ surface coating. The dynamic rheology results in Figure 3j show that the 40 vol % slurry transitioned from the predominantly elastic behavior seen in Figure 2h to viscoelastic behavior, indicating markedly improved flowability. Although the 50 vol % slurry still shows G’ > G”, reflecting retained elasticity, both moduli are reduced by 1–2 orders of magnitude, suggesting enhanced particle mobility. This improvement is expected to facilitate more effective magnetization and particle alignment during film formation. Furthermore, the cross-sectional image of the dried MZ@SiO_2_/GPTMS/epoxy film (Figure 3k) reveals substantially improved particle adhesion within the epoxy matrix, further demonstrating the benefit of the SiO_2_ surface modification.

### 3.4 Magnetorheological Properties

According to electromagnetic theory, well-aligned magnetic particles are essential for achieving optimal magnetic permeability.^47^ To evaluate whether the better-dispersed MZ@SiO_2_/GPTMS/epoxy slurry facilitates particle alignment more effectively than the ferrite/GPTMS/epoxy slurry, their rheomagnetic properties were analyzed prior to drying. As shown in Figure 4a, the slurry containing 50 vol % unmodified MnZn ferrite powder was subjected to a magnetic field (0.1–0.4 T) perpendicular to the flow direction during rheological measurements. The shear stress required to induce flow increased with both shear rate and magnetic field strength. A similar trend is observed for the slurry with 50 vol % MZ@SiO_2_ powder (Figure 4b), but the increase in shear stress with magnetic field is more pronounced.

**Figure 4:**
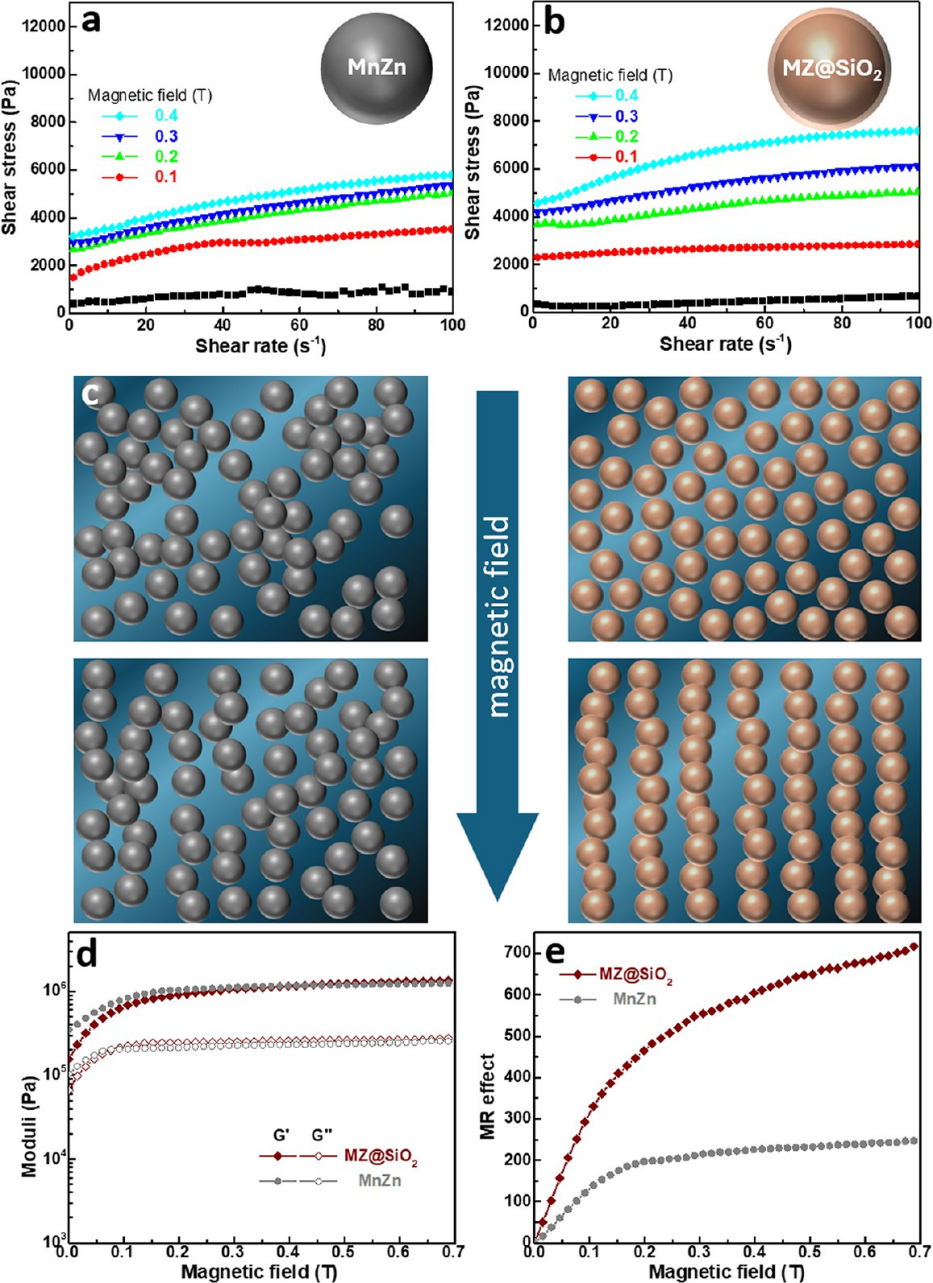
Shear stress as a function of shear rate under varying magnetic field strengths for 50 vol % slurries of (a) MnZn ferrite and (b) MZ@SiO_2_ in epoxy with 5 wt % GPTMS. (c) Schematics illustrating the magnetic-field-induced alignment of MnZn ferrite and MZ@SiO_2_ particles in the slurries. (d) G′ and G″ moduli as functions of magnetic field strength, and (e) MR effect of the 50 vol % slurries used in (a) and (b).

To quantify this behavior, the magnetorheological data were fitted using the Herschel-Bulkley model (eq 3) to extract the yield stress (τ_0_), with results summarized in Table 2.

$$\tau =\tau_{0}+k{\mathrm{\gamma ̇}}^{n}$$ 3

where τ is the shear stress, *k* is the consistency index, γ̇ is the shear rate, and n is the flow index. The increase in τ_0_ with magnetic field strength is attributed to enhanced particle alignment and the formation of more robust chain structures,^7,48^ which resist flow more strongly. Accordingly, a higher τ_0_ is needed to disrupt these structures and initiate flow. Notably, the MZ@SiO_2_-based slurries exhibited consistently higher τ_0_ values at all magnetic field strengths, indicating that the modified particles align more readily and form stronger chains. This behavior can be attributed to the improved dispersion and smaller size of the MZ@SiO_2_ particles than the unmodified MnZn ferrite in the GPTMS/epoxy matrix, which facilitates more effective alignment, as illustrated in Figure 4c.

**Table 2:** Yield Stress (τ_0_) of Magnetic Slurries with 50 vol% Magnetic Powder and GPTMS (5 wt % Based on the Powder Mass) under Varying Applied Magnetic Field Strengths

	τ_0_ (Pa)	
magnetic field strength (T)	MnZn ferrite	MZ@SiO_2_
0	307	259
0.1	1620	1697
0.2	2219	3362
0.3	2524	3908
0.4	2550	4402

Additionally, the G’ and G” values of both slurries were measured under varying magnetic field strengths, as shown in Figure 4d. For both systems, G’ and G” initially increase with the magnetic field, then gradually level off. While the G” values of the two slurries are relatively similar, their G’ responses differ, with the MZ@SiO_2_/GPTMS/epoxy slurry showing a more notable increase in G’ compared to the ferrite/GPTMS/epoxy slurry. Using eq 4 and the G’ values from Figure 4d, the magnetorheological (MR) effect can be further obtained via

$$\mathrm{MR}\;\mathrm{effect}=\frac{G_{H}^{\prime }-G_{0}^{\prime }}{G_{0}^{\prime }}\times 100\%$$ 4

where *G*_*H*_’ is storage modulus at a specific magnetic field strength, and *G*_0_’ is storage modulus of the slurry when there is no external magnetic field. An MR effect of zero indicates no particle chain formation, whereas higher values signify stronger MR effects and more developed field-induced chain structures.

As shown in Figure 4e, the MZ@SiO_2_/GPTMS/epoxy slurry exhibits significantly higher MR values than the ferrite/GPTMS/epoxy slurry across all magnetic field strengths, indicating a stronger MR response. This result is consistent with Figure 4a,b, where improved particle dispersion in the MZ@SiO_2_-based slurry facilitates easier alignment under the magnetic field. As illustrated in Figure 4c, large agglomerates in the unmodified ferrite slurry likely experience greater resistance to magnetic-field-induced rearrangement.

### 3.5 Effect of Particle Arrangements on Magnetic Properties

After confirming the superior magnetorheological properties of the MZ@SiO_2_/GPTMS/epoxy slurry, it was used in subsequent experiments to fabricate magnetic composite films. Figure 5a shows the top-view image of a 100-μm-thick MZ@SiO_2_/GPTMS/epoxy film containing 50 vol % powder, where the MZ@SiO_2_ particles are uniformly dispersed within the epoxy matrix. To facilitate the alignment of magnetic particles, the slurry was cast and dried under a magnetic field. The resulting top-view image after drying is shown in Figure 5b, where the MZ@SiO_2_ particles exhibit clear alignment, forming chain-like structures along the field direction.

**Figure 5:**
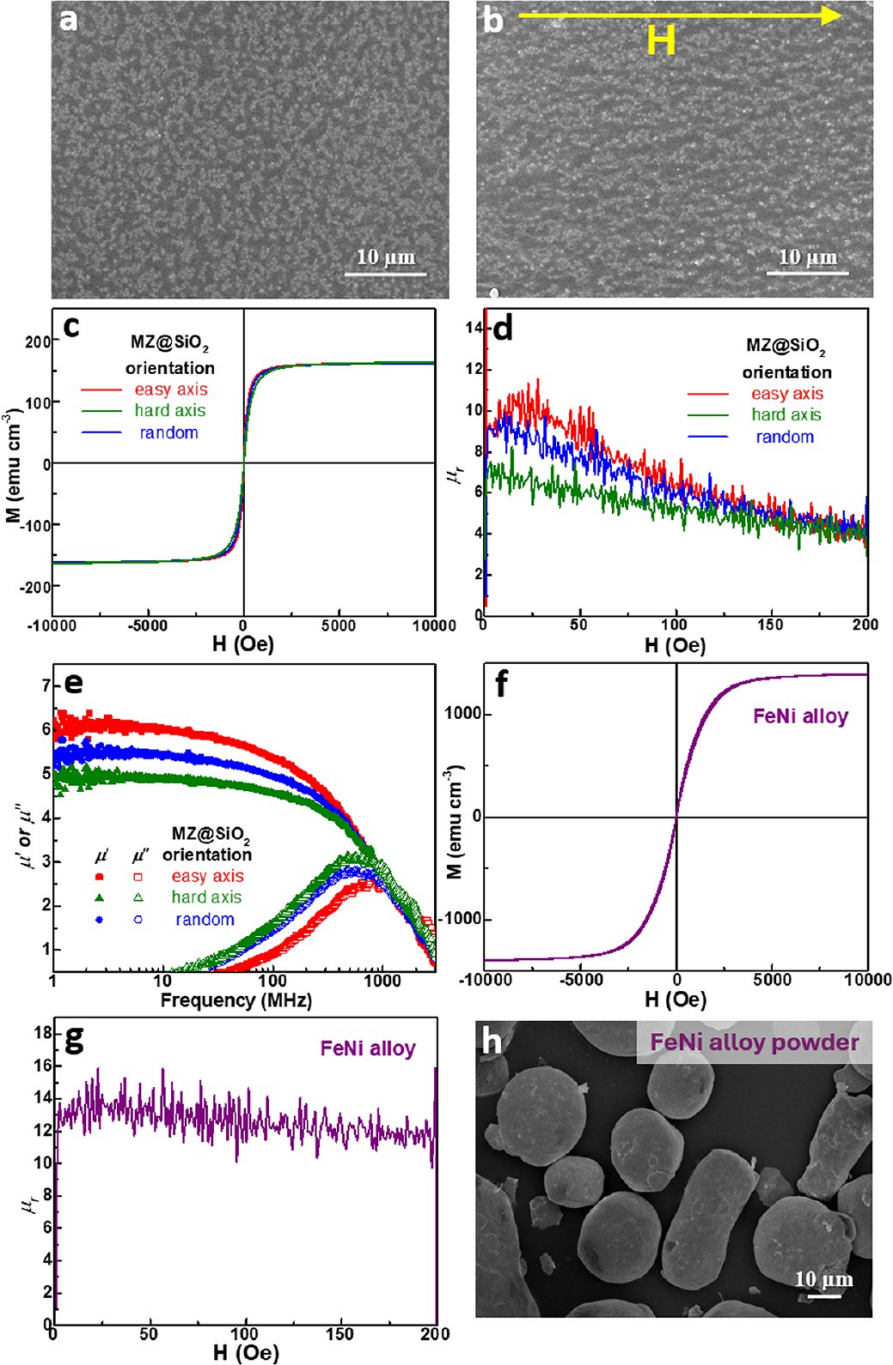
SEM images (top view) of the MZ@SiO_2_/GPTMS/epoxy film (50 vol %) (a) before and (b) after particle alignment using an external magnetic field of 0.04 T. (c) M-H loop, (d) μ_*r*_, and (e) μ′ and μ″ as functions of frequency for the films in (a) and (b) with particles aligned in different orientations. (f) M-H loop, (g) μ_*r*_, and (h) SEM image of as-received FeNi alloy powder. Note that μ′ and μ″ are dimensionless quantities. All magnetic and frequency-dependent measurements were repeated on three independently fabricated samples, yielding consistent results with negligible variation.

To elucidate the impact of particle alignment on the magnetic performance of the composite films, the two films from Figure 5a and 5b were evaluated for their magnetization behavior. Notably, the alignment of particles introduces anisotropy in the film, with the direction parallel to the magnetic field defined as the easy axis and the perpendicular direction as the hard axis. The magnetic properties of the aligned film along both directions were separately analyzed and compared to those of the nonaligned (denoted as “random”) film. As shown in Figure 5c, all three films display similar Ms values, suggesting that particle arrangement does not influence the final magnetization level. However, the magnetization rises more quickly with the applied magnetic field along the eas*y*-axis direction, followed by the random and hard-axis films, as clearly illustrated in the magnified inset view. This result suggests that particle alignment facilitates the approach to Ms under lower magnetic fields and demonstrates the anisotropic magnetic response induced by alignment. Furthermore, using the data from Figure 5c and applying eq 1, we calculated the μ_*r*_ of the three films, as presented in Figure 5d. The results show that the film with particles aligned along the easy axis exhibits the highest μ_*r*_, followed by the film with particles randomly oriented, with particles aligned along the hard axis displaying the lowest μ_*r*_. This trend reflects the influence of particle alignment on the magnetic response, where alignment along the easy axis facilitates greater magnetic permeability.

It is acknowledged that the μ_*r*_ can be derived from M-H data, which provides information on the material’s static or low-frequency magnetic response.^49^ Although this method is straightforward and useful for understanding intrinsic magnetic properties under quasi-static conditions, measurements using a VNA are preferable to obtain permeability values that better represent the material’s behavior under practical operating conditions, especially at high frequencies relevant to RF and microwave applications. VNA characterization measures the frequency-dependent μ_*r*_, including both magnetic losses and dispersion effects, providing a more accurate and comprehensive evaluation for practical applications. The real component (μ’) of μ_*r*_ corresponds to magnetic energy storage, whereas the imaginary component (μ”) reflects energy dissipation. As shown in Figure 5e, VNA measurements are conducted to further characterize the μ’ and μ” of the films with three different particle orientations. The results follow the same trend observed in Figure 5d: the film with particles aligned along the easy axis exhibits the highest μ’, followed by the randomly oriented film, and then the hard-axis. However, it is noteworthy that the μ’ values obtained from the VNA are consistently lower than those calculated from the M-H data. This discrepancy is likely due to frequency-dependent effects and magnetic losses, which are captured in the VNA measurements but not accounted for in the quasi-static M-H analysis. Nonetheless, the agreement in trend between both methods reinforces that particle alignment plays a crucial role in determining the magnetic permeability of the composite films, with eas*y*-axis alignment providing the most favorable magnetic response.

### 3.6 Hybrid with FeNi Alloy for Enhanced Magnetic Properties

In addition to MnZn ferrites, FeNi alloy is a promising material for high-performance magnetic applications due to its significantly higher Ms (Figure 5f) and μ’ (Figure 5g) compared to MnZn ferrite. However, a major challenge arose when preparing FeNi-based composite film: its relatively large particle size (30–50 μm) (Figure 5h) made it difficult to align the particles within the thin film (Figure S6, Supporting Information). The limited mobility during the short alignment window before the solvent evaporation hindered the creation of a well-ordered structure. To overcome this, a hybrid film was developed. It incorporated 25 wt % FeNi alloy into the MZ@SiO_2_ composite film, creating the MZ@SiO_2_/FeNi/epoxy film. It is noted that the FeNi alloy content of 25 wt % was selected based on preliminary processing trials that identified it as the highest loading that could be uniformly incorporated into the composite matrix while maintaining satisfactory film uniformity and castability. Higher FeNi fractions (>25 wt %) led to particle stacking and disruption of the film microstructure due to the large FeNi particle size, preventing reliable formation of continuous magnetic pathways. Thus, 25 wt % represents the optimal balance between magnetic enhancement and fabrication feasibility for the hybrid film system. This approach successfully combined the high magnetic strength of FeNi with the excellent dispersibility of the MnZn ferrite particles. The resulting film exhibited enhanced Ms (Figure 6a) and μ’ (Figure 6b) compared to the original MZ@SiO_2_ film (Figure 5c and 5e), confirming the effectiveness of this design (Figure S7, Supporting Information). Furthermore, the limited FeNi content, along with the insulating SiO_2_ shell on the ferrite particles, effectively suppressed eddy current losses, ensuring a high μ’ across a wide frequency range (Figure 6b compared with Figure 5e).

**Figure 6:**
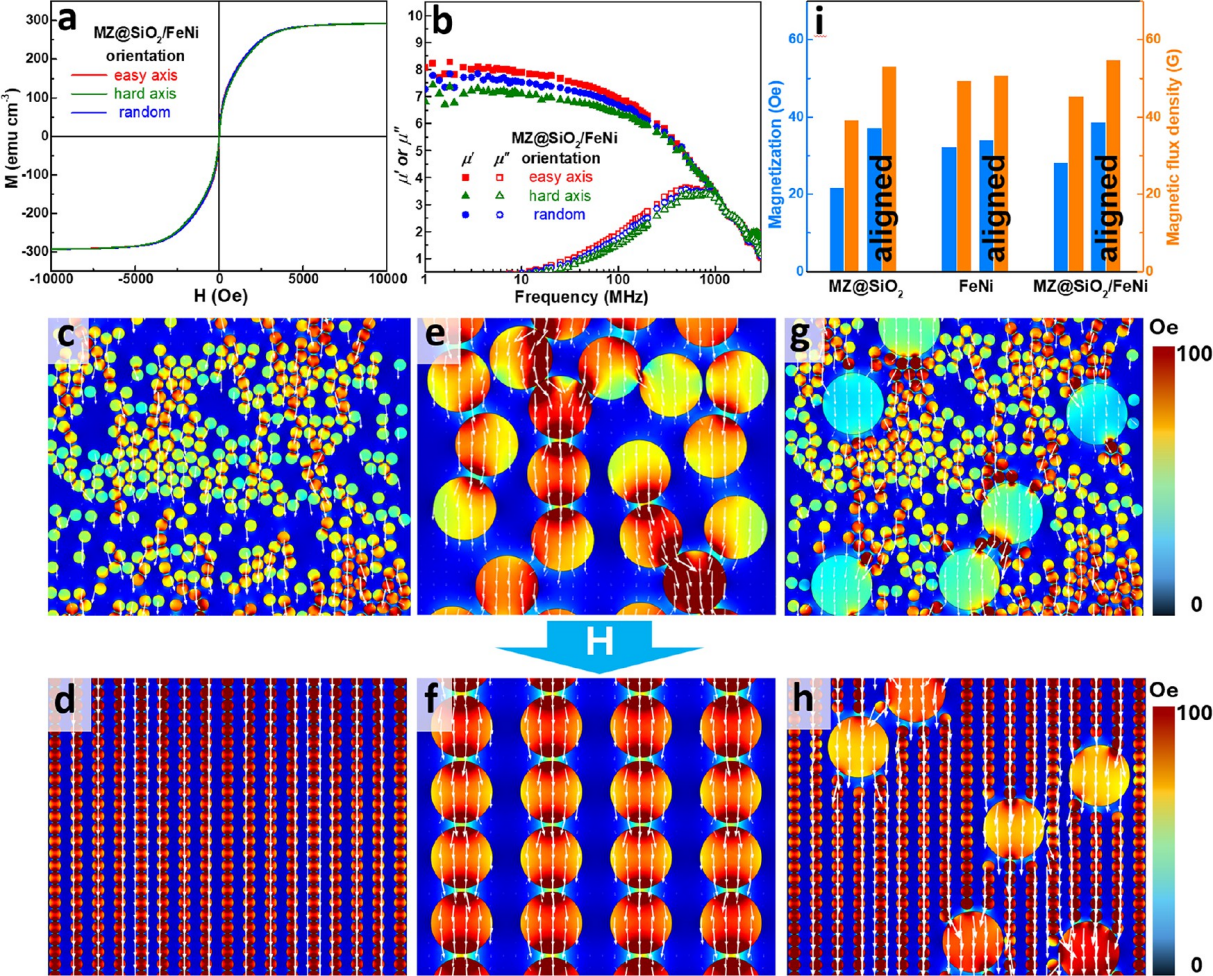
(a) M-H loop and (b) μ′ and μ″ as functions of frequency for the hybrid film composed of MZ@SiO_2_ and FeNi (3:1 v/v) in GPTMS/epoxy. (c–h) FEM simulations of magnetization for magnetic films: (c, d) MnZn ferrite, (e, f) MZ@SiO_2_, and (g, h) hybrid of MZ@SiO_2_ and FeNi alloy (3:1 v/v) in GPTMS/epoxy, shown (c, e, g) before and (d, f, h) after particle alignment. (i) Simulated magnetization and magnetic flux density as functions of particle arrangement corresponding to (c–h). Note that μ′ and μ″ are dimensionless quantities. The experimental magnetic measurements in (a) and (b) were verified across three independently fabricated samples, showing negligible sample-to-sample variation.

It may seem counterintuitive that a composite film containing hard-to-align FeNi alloy particles would exhibit enhanced magnetic properties. However, the observed improvement can primarily stem from the MnZn ferrite component, whose magnetic behavior is expected to be significantly more sensitive to particle alignment than that of FeNi. This distinction may arise from their respective magnetic domain structures: the smaller (∼1 μm) MnZn ferrite particles likely fall within the single-domain regime for spinel ferrites,^50^ where magnetization changes are governed by coherent rotation, an orientation-dependent mechanism. In contrast, the larger (30–50 μm) FeNi alloy particles should have surpassed the critical size for single-domain behavior, displaying multidomain structures where magnetization is primarily driven by domain wall motion,^51^ which is less sensitive to particle orientation. This theoretical understanding can be supported by FEM simulation results (Table S1, Supporting Information). For MnZn ferrite-based films, a clear enhancement in magnetization is observed after alignment, as shown by comparing Figure 6c and 6d. In contrast, FeNi-based films show a less pronounced difference before and after alignment (Figure 6e and 6f), indicating a weaker dependence on orientation. Simulations of the hybrid MZ@SiO_2_/FeNi (25%) composite films, before and after alignment (Figure 6g and 6h), show further improvement, exceeding that of both individual systems. For easy comparison, Figure 6i presents the magnetization and magnetic current density of all three systems, both aligned and nonaligned, confirming that the alignment effect agrees well with theoretical predictions. Notably, the superior performance of the hybrid film arises mainly from the well-aligned MnZn ferrite particles, whose magnetic properties are highly orientation-dependent, rather than the FeNi alloy particles.

### 3.7 Inductor Performances

To further evaluate the magnetic properties, such as inductance and quality factor, of the fabricated composite films, the films were integrated into an air-core planar inductor, with the preparation procedure illustrated in Figure 7a. The fabrication of the composite film-covered Cu coil inductor begins with thermal transfer of a printed spiral pattern from PCB heat transfer paper onto a copper-clad laminate, followed by etching with sodium persulfate to remove unwanted copper and form the inductor geometry, after which a magnetic composite slurry is tape-cast onto the patterned surface to create a uniform 100 μm-thick magnetic film. Notably, to investigate the anisotropic effects of the composite films, the Cu coil was designed with a rectangular shape, measuring 2 cm in length and 1 cm in width. Additionally, photographs (Figure S8, Supporting Information) and corresponding videos (Videos S1 and S2, Supporting Information) demonstrate the excellent flexibility of the fabricated hybrid film composed of MZ@SiO_2_ and FeNi alloy (3:1 v/v), visually confirming its mechanical robustness under bending and twisting. A tensile test was also performed to evaluate its mechanical strength, and the resulting stress–strain curve is presented in Figure S9 (Supporting Information).

**Figure 7:**
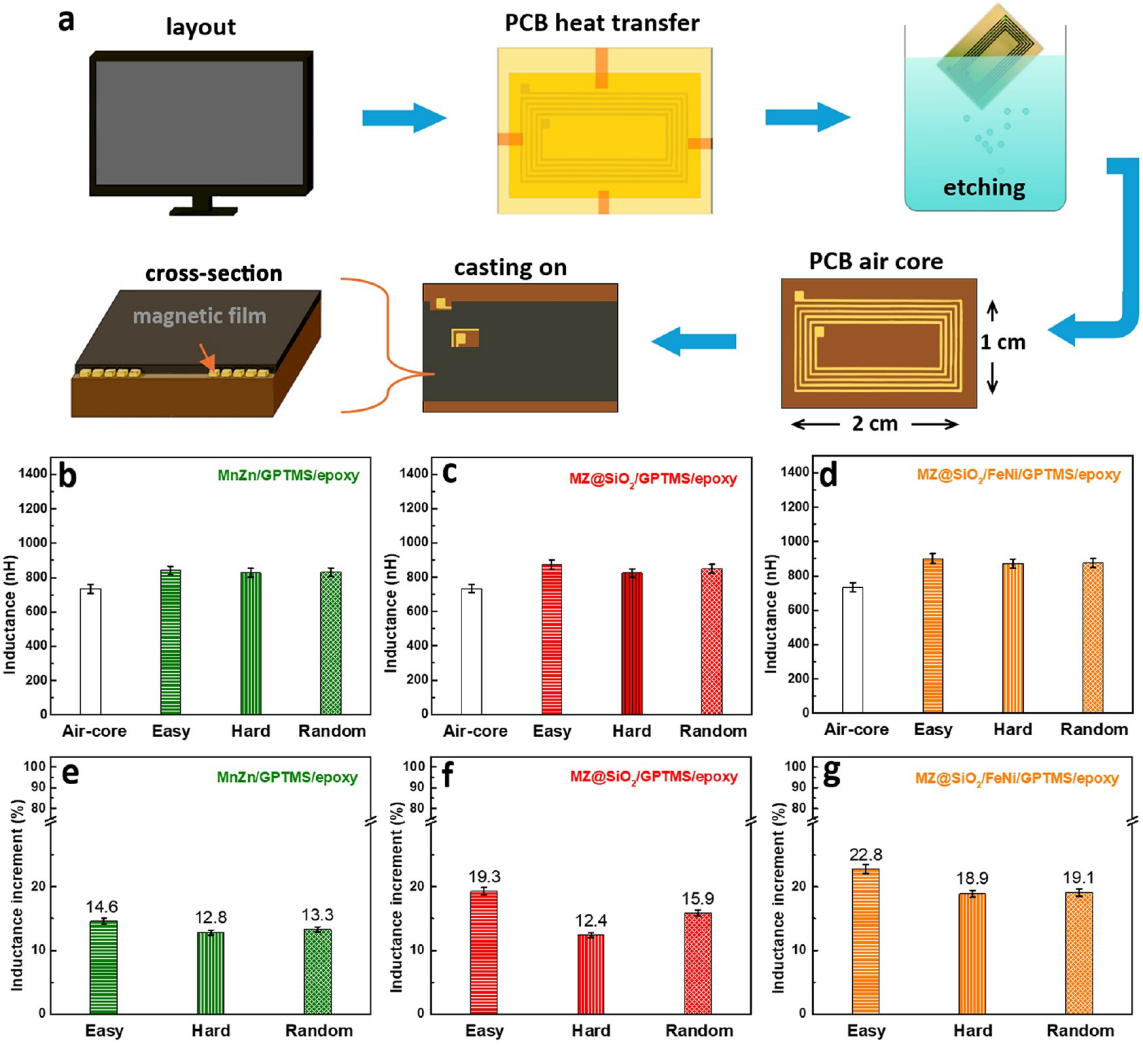
(a) Schematic illustrating the fabrication process of a flexible magnetic film inductor. (b–d) Inductance and (e–g) inductance enhancement of a planar Cu air-core inductor integrated with magnetic films composed of (b, e) MnZn ferrite, (c, f) MZ@SiO_2_, and (d, g) a hybrid of MZ@SiO_2_ and FeNi alloy (3:1 v/v), with magnetic particles aligned in different orientations. Error bars represent the statistical variation from five to six independently fabricated samples.

Figure 7b-d presents the inductance (L) values measured at 1 MHz for various anisotropic magnetic composite films containing 50 vol % magnetic filler integrated with air-core copper inductors. The inductance of the bare air-core copper coil (L = 733.93 nH) is also included as a reference. Figure 7b shows the inductance values for inductors integrated with ferrite/GPTMS/epoxy films having different particle alignment orientations, and the corresponding inductance gains compared to the air coil are summarized in Figure 7e. Although all composite-integrated inductors exhibit higher inductance values than the bare air coil, the influence of particle alignment direction on inductance improvement is not significant. This could be attributed to the limited enhancement of interfacial bonding between MnZn ferrite particles and the epoxy matrix by GPTMS (Figure 2g). Although GPTMS improves particle dispersion of MnZn ferrite, insufficient interfacial adhesion leads to discontinuous magnetic flux pathways, hindered flux transmission, and reduced electromagnetic coupling, ultimately suppressing inductance performance.

In contrast, Figure 7c and 7f correspond to magnetic films based on MZ@SiO_2_ particles, where the ferrite surface is modified with SiO_2_. A clear improvement is observed in both the absolute inductance values and the dependence on particle orientation compared to the ferrite/GPTMS/epoxy system in Figure 7b. Especially under eas*y*-axis alignment, the magnetic particles tend to form chain-like structures with excellent magnetic permeability (Figure 5), promoting continuous magnetic flux paths, increasing magnetic flux density, and reducing energy losses. This enhances inductive efficiency and energy storage capability.^47^ These results demonstrate the significance of SiO_2_ surface modification in enhancing interfacial compatibility between the filler and polymer matrix, a crucial factor in achieving superior device performance. Furthermore, Figure 7d and 7g show the results for films composed of MZ@SiO_2_/FeNi hybrid powders, which clearly outperform the previous two systems. Under eas*y*-axis alignment, the inductance exceeds 900 nH, representing a ∼ 22.8% increase over the air-core inductor.

Additionally, the quality factor (Q) was used to evaluate the performance of the inductor coils. The quality factor represents the ratio of stored energy to dissipated energy at a specific frequency, and is defined by the following equation:

Q=2πfL/R 5

where f is the frequency (Hz) and R is the equivalent series resistance (Ω). Since R reflects the resistive losses in the system, it plays a crucial role in determining the Q factor and, consequently, the energy efficiency of the component. Figure 8a-c shows the frequency-dependent Q factors for inductor coils integrated with magnetic thin films of different anisotropies (easy, hard, and random), as well as for a reference air-core coil. Figure 8a presents the Q factor of the coil integrated with the ferrite/GPTMS/epoxy film. As the frequency increases, both the integrated and air-core coils exhibit a rising Q trend, primarily due to improved inductive response and changes in impedance at higher frequencies. Across the entire frequency range, the integrated coil (green curve) consistently demonstrates higher Q values than the air-core coil (blue curve), confirming that the addition of magnetic material enhances energy storage efficiency while reducing losses. The improvement is especially evident in the high-frequency region (∼10^6^ Hz), where the Q value of the eas*y*-axis film reaches 8.11 compared to 7.08 for the air-core coil, an increase of approximately 14.5%. Furthermore, Figure 8d compares the Q-factor enhancement resulting from films with different particle alignment orientations. Despite the intended anisotropy, the Q values of the coils integrated with ferrite/GPTMS/epoxy films exhibit only minor differences among the three alignment conditions. This minimal variation suggests that the magnetic anisotropy was not effectively translated into enhanced performance. A likely explanation lies in the limited interfacial bonding between the MnZn ferrite particles and the epoxy matrix, as aforementioned. Consequently, magnetic flux propagation may be hindered regardless of alignment orientation, resulting in similarly limited Q factor enhancement.

**Figure 8:**
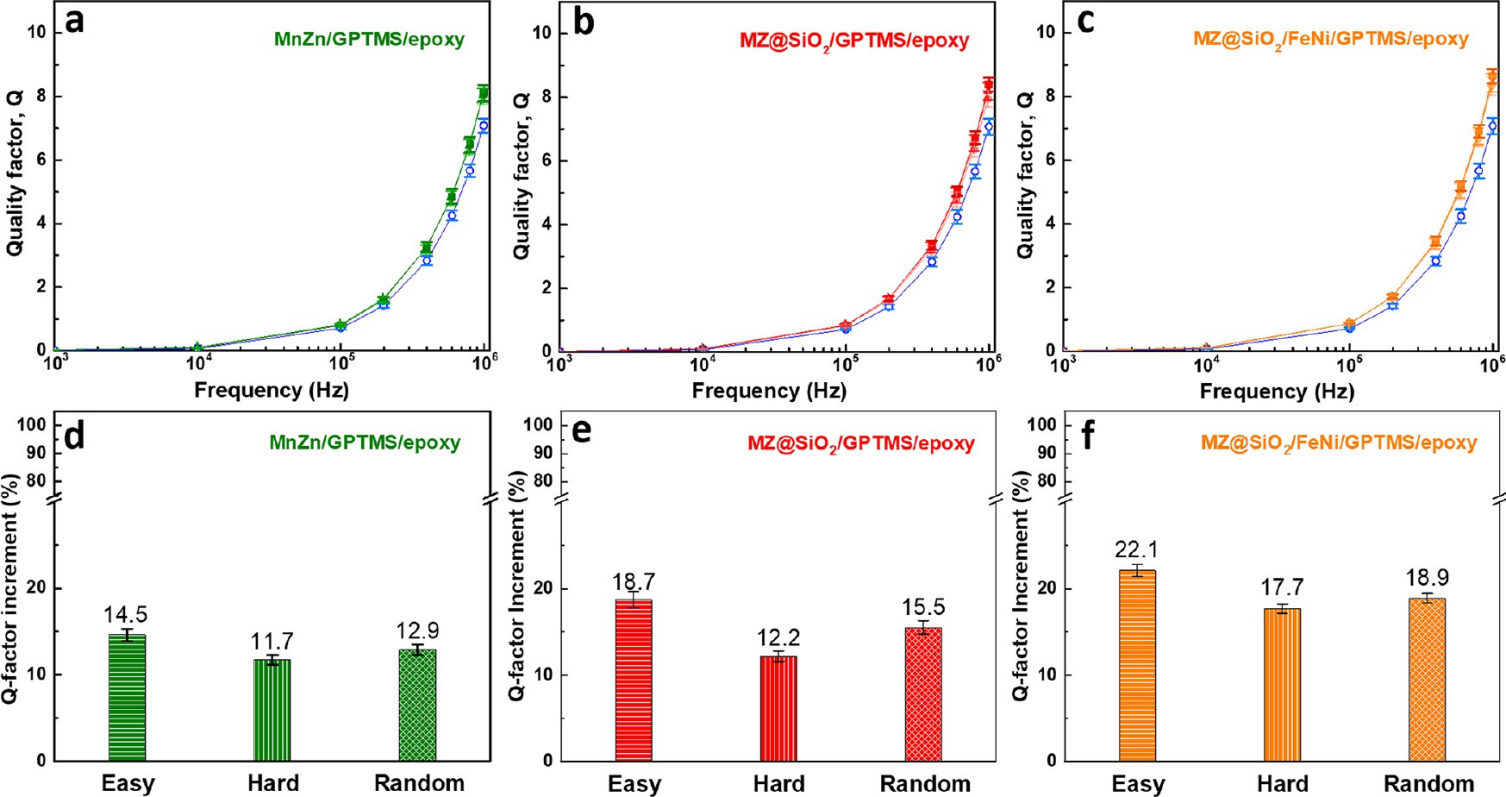
(a–c) Q-factor and (d–f) Q-factor enhancement of a planar Cu air-core inductor integrated with magnetic films composed of (a, d) MnZn ferrite, (b, e) MZ@SiO_2_, and (c, f) a hybrid of MZ@SiO_2_ and FeNi alloy (3:1 v/v), with magnetic particles aligned in different orientations. Error bars represent the statistical variation from five to six independently fabricated samples.

Figure 8b presents the frequency-dependent Q values of inductor coils integrated with MZ@SiO_2_/GPTMS/epoxy magnetic films. Compared to the ferrite/GPTMS/epoxy system shown in Figure 8a, the overall Q values in Figure 8b are markedly higher, particularly in the high-frequency range. This enhancement is primarily attributed to the SiO_2_ surface modification, which improves the dispersion of magnetic particles and enhances interfacial compatibility with the epoxy matrix. As a result, magnetic coupling is more efficient, leading to better energy storage and reduced electromagnetic loss. The influence of magnetic particle alignment becomes even clearer in Figure 8e, which compares the Q-factor improvements (relative to the air-core coil) across three alignment conditions. In contrast to the MnZn ferrite system (Figure 8d), where the alignment-induced difference is minimal, Figure 8e exhibits a more pronounced anisotropic response. The eas*y*-axis alignment yields the highest Q enhancement, while the hard-axis alignment is the lowest, with the random condition falling in between. This observation supports the hypothesis that SiO_2_ modification not only improves interfacial bonding but also enables more effective translation of particle alignment into functional magnetic anisotropy, facilitating better magnetic flux conduction along the aligned direction.

Figure 8c introduces a hybrid composite system consisting of 25 vol % FeNi alloy and 75 vol % MZ@SiO_2_, and the corresponding Q-factor behavior is similar in its anisotropy to that of Figure 8d and 8e. As previously discussed, this film remains sensitive to alignment because of the dominant contribution from the orientation-dependent magnetization of the MZ@SiO_2_ component. In this hybrid system, the FeNi particles enhance the overall permeability and reduce loss due to their high saturation magnetization, but contribute less directly to alignment effects. Figure 8f quantitatively compares the Q-factor enhancement rates under different alignment orientations for the hybrid system. The results mirror the trends seen in Figure 8c and confirm that alignment remains a key factor, even in composite systems containing hard-to-align magnetic fillers. The successful transmission of anisotropic magnetic behavior through aligned MnZn ferrite highlights the importance of multiphase design, where each magnetic component plays a distinct but complementary role. To provide a clearer quantitative comparison, Table 3 summarizes the key magnetic properties, including saturation magnetization, relative permeability, real part of permeability, Q-factor improvement, and inductance enhancement, for the MnZn ferrite, MZ@SiO_2_, and hybrid MZ@SiO_2_/FeNi composite films that were prepared without particle alignment during film fabrication. This comparison demonstrates the overall improvement in magnetic performance achieved through SiO_2_ surface modification and FeNi incorporation, elucidating the distinct yet complementary contributions of each component within the composite system.

**Table 3:** Comparison of Key Magnetic Properties for Unaligned Composite Films of MnZn Ferrite, MZ@SiO_2_, and Hybrid MZ@SiO_2_/FeNi

**sample**	**Ms (emu cm** ^**–3**^ **)**	**μ**_*r*_ **(by VSM)**	**μ’ (by VNA)**	**Q-factor improvement (%)**	**L improvement (%)**	**remarks**
MnZn ferrite/GPTMS/epoxy	165	7	5	14.5	14.6	• poor dispersion
						• limited interfacial bonding
MZ@SiO_2_/GPTMS/epoxy	162	9	6	18.7	19.3	• improved dispersion
						• good interfacial compatibility
						• pronounced anisotropic response
hybrid MZ@SiO_2_/FeNi /GPTMS/epoxy	293	9.5	8	22.1	22.8	• enhanced Ms, μ_r_, μ′ from FeNi
						• anisotropy dominated by MZ@SiO_2_ alignment
						• eddy losses suppressed by SiO_2_ shell

To further contextualize the magnetic and device performance, the results of this work were benchmarked against reported flexible and planar magnetic inductors based on soft magnetic composites.^6–18^ As summarized in these studies, such inductors typically exhibit inductance values ranging from several nanohenries to a few microhenries and Q-factors between 5 and 50 at MHz frequencies, depending on magnetic loss and conductor resistance. The present MZ@SiO_2_/FeNi hybrid film exhibits an inductance enhancement of approximately 23% and a Q-factor of approximately 8 at 1 MHz, which falls within the upper range of flexible polymer-based systems, while being achieved through a low-temperature, epoxy-based fabrication route. This benchmarking underscores the competitiveness and practical relevance of the present composite design for next-generation electromagnetic device applications.

## 4 Conclusions

In this study, proper thermal annealing was shown to eliminate undesirable α-Fe_2_O_3_ impurities in the MnZn ferrite powders and promote the growth of magnetically active crystalline phases. Specifically, annealing at 600 °C in an Ar atmosphere optimized the magnetic properties while preventing excessive agglomeration or sintering, thereby maintaining the processability of the powders. To enhance the interfacial compatibility between the MnZn ferrite particles and the epoxy matrix, GPTMS was employed as the coupling agent. This treatment allowed the filler loading to increase from 40 vol % to 50 vol % without compromising dispersion quality. In addition, the Stöber method was adopted to coat the MnZn ferrite particles with a SiO_2_ shell (MZ@SiO_2_), which enhanced dispersion stability and improved the rheological behavior of the magnetic slurry. Magnetorheological measurements indicated that MZ@SiO_2_ powders readily formed chain-like structures under an external magnetic field, leading to a stronger MR effect during film fabrication. Cross-sectional microscopy further showed that, with GPTMS treatment, MZ@SiO_2_ particles established better interfacial bonding with the epoxy matrix and formed more continuous and aligned particle chains. Magnetic characterization via VSM revealed that MZ@SiO_2_-based films exhibited clear magnetic anisotropy. At identical filler loading, magnetic composite films aligned along the easy axis, hard axis, and those with random orientation showed distinct differences in magnetic permeability. Frequency-domain analysis using a VNA demonstrated that MZ@SiO_2_ films maintained good magnetic permeability of ∼ 6 at frequencies <100 MHz. Hybrid films containing 25 vol % FeNi alloy and 75 vol % MZ@SiO_2_ were also examined. FEM simulations showed that the magnetic properties of films with larger FeNi alloy particles are relatively insensitive to their orientation. Magnetic measurements further revealed that these hybrid films exhibit higher saturation magnetization and a permeability of ∼ 8, surpassing the performance of MZ@SiO_2_-only films. These results confirm the energy-storage benefits of incorporating FeNi. Regarding device performance, the inductor coil integrated with the hybrid MZ@SiO_2_/FeNi/epoxy film showed increased inductance and Q value, emphasizing the potential of this film for magnetic storage applications to support the development of next-generation magnetic composites that combine high permeability, low magnetic losses, and tunable anisotropy.

## Supplementary Material







## Notes

Parameters in Table S1 for FEM simulation; SEM images and SEM-EDS mapping images of ferrite powders and magnetic thin films; XPS and elemental depth profiles of SiO_2_-coated MnZn ferrite particles; TEM image of SiO_2_-coated MnZn ferrite particle; magnetic and frequency-dependent properties of various ferrite composite films; photographs and tensile stress–strain curves of the hybrid MZ@SiO_2_/FeNi composite film (PDF)
Bending video (MP4)
Twisting video (MP4)

## Abbreviations

EMI: electromagnetic interference
RF: radio frequency
Ms: saturation magnetization
μ_*r*_: relative permeability
μ′: real component of μ_*r*_
μ″: imaginary component of
μ_*r*_: Q-factor, quality factor
GPTMS: 3-glycidyloxypropyltrimethoxysilane
APTES: 3-aminopropyltriethoxysilane
PCB: printed-circuit-board
SEM: scanning electron microscope
XPS: X-ray photoelectron spectroscopy
XRD: X-ray diffraction
*G*′: storage modulus
*G*″: loss modulus
FEA: finite element analysis
η: viscosity
MZ@SiO_2_: SiO_2_-coated MnZn ferrite
MR effect: magnetorheological effect
MZ@SiO_2_/FeNi: 25 vol % FeNi hybrided with 75 vol % MZ@SiO_2_ in epoxy
L: inductance
