# Supplementary material for: Flexible Ferrite Magnetic Composite Films for Electromagnetic Applications
Source: ACS Mater Au. 2025 Dec 12;6(2):390–405. doi: 10.1021/acsmaterialsau.5c00180 (PMC12983101; doi:10.1021/acsmaterialsau.5c00180)
Supplement: Supplementary file 1 [file mg5c00180_si_001.pdf]

Supporting Information

Flexible Ferrite Magnetic Composite Films for Electromagnetic Applications

Jui-Yang Hsu, Chih-Huang Lai, and Chia-Chen Li*

Department of Materials Science and Engineering, National Tsing Hua University, Hsinchu 30013, Taiwan

*Corresponding author. E-mail: cc.li@mx.nthu.edu.tw

Supplementary Table

Table S1 Parameters used in FEM simulations for the magnetization of magnetic films.

Object	Parameter (unit)	Value
NdFeB magnet	radius, height (cm)	0.75, 7.5
	temperature (K)	293.15
	pressure (atm)	1
	magnetic scalar potential (A)	0
	surface magnetic field strength (A m^{-1})	5×10^4
magnetic bar	length, width, height (cm)	8, 0.75, 1.5
	temperature (K)	293.15
	pressure (atm)	1
	magnetic scalar potential (A)	0
	relative permeability	5×10^4
epoxy substrate	length, width, height (cm)	6.5, 5.5, 1
	temperature (K)	293.15
	pressure (atm)	1
	magnetic scalar potential (A)	0
	relative permeability	1
MnZn ferrite	radius (μm)	10
	temperature (K)	293.15
	pressure (atm)	1
	magnetic scalar potential (A)	0
	relative permeability	10
FeNi alloy	radius (μm)	50
	temperature (K)	293.15
	pressure (atm)	1
	magnetic scalar potential (A)	0
	relative permeability	15

For the numerical simulations, calculations were performed using the magnetic fields with no currents (mfnc) interface in the AC/DC module.

Supplementary Figures

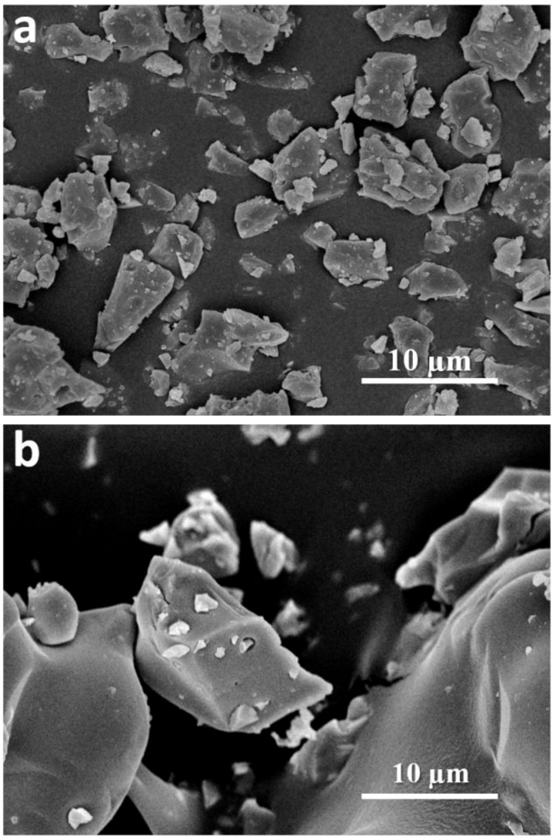

Figure S1. SEM images of MnZn ferrite powder annealed under Ar at (a) 800 ^{o}C and (b) 1000 ^{o}C, followed by mechanical grinding.

The results in Figure S1 indicate that lab-scale mechanical grinding is ineffective in reducing the annealed MnZn ferrite powder to smaller and more uniformly sized particles.

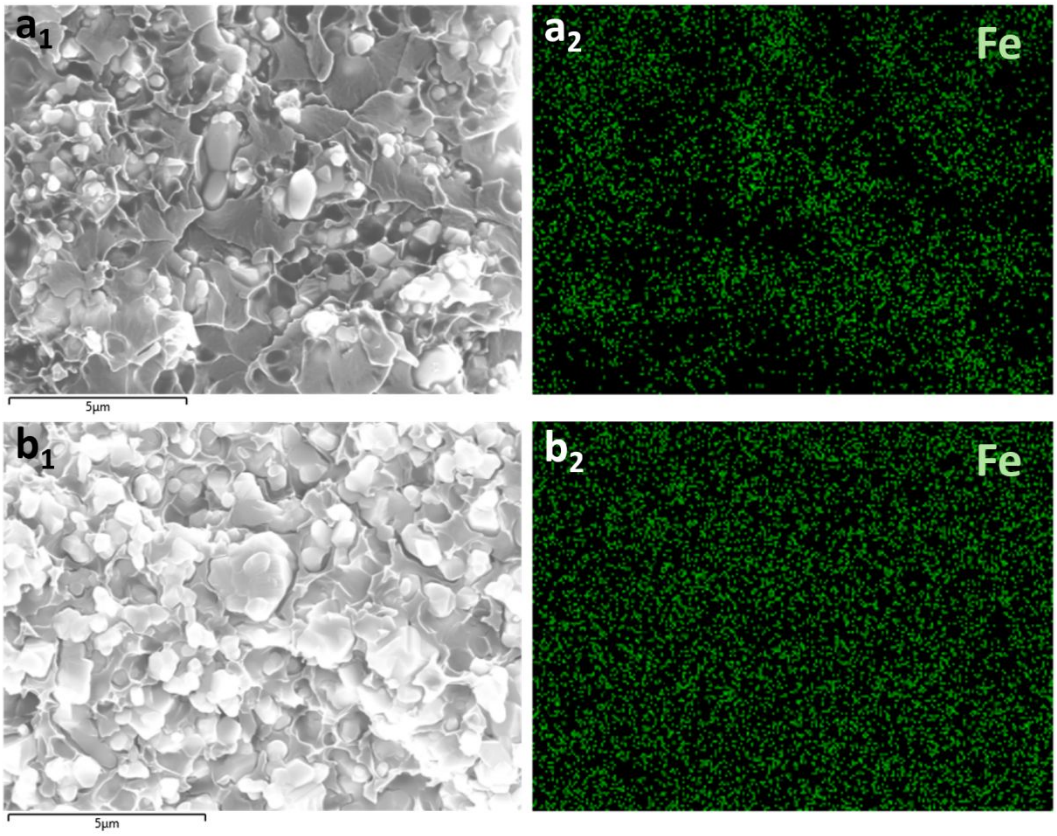

Figure S2. SEM images and corresponding EDS mappings of Fe element for the cross-sections of magnetic films: (a_1,a_2) 40 vol% MnZn ferrite in epoxy, and (b_1,b_2) 50 vol% MnZn ferrite in epoxy with 5 wt% GPTMS (relative to ferrite mass) added.

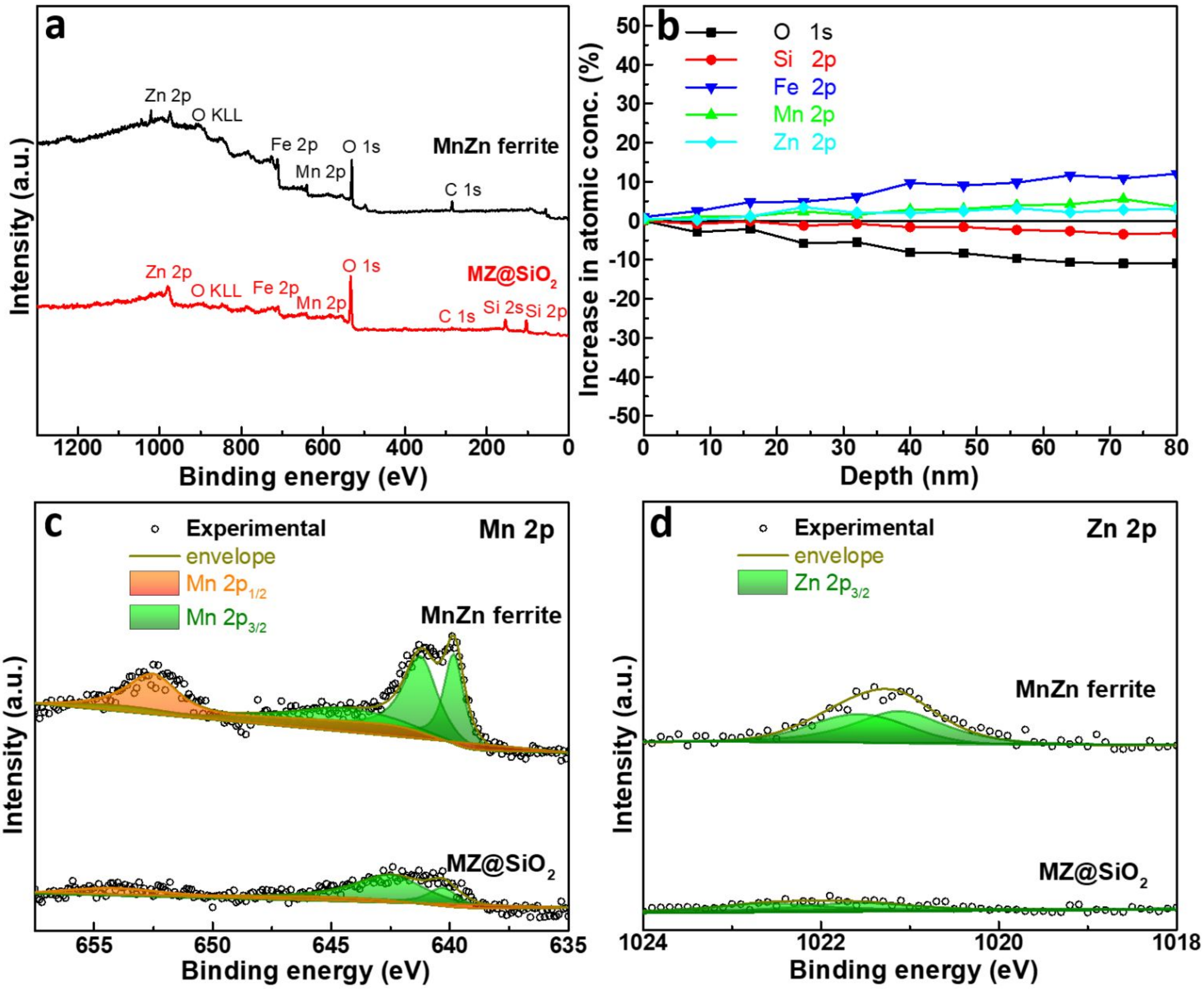

Figure S3. (a) XPS survey spectra of MnZn ferrite and MZ@SiO_2. (b) XPS depth profile analysis of elemental compositions for MZ@SiO_2. High-resolution spectra showing the chemical states of (b) Mn 2p and (c) Zn 2p.

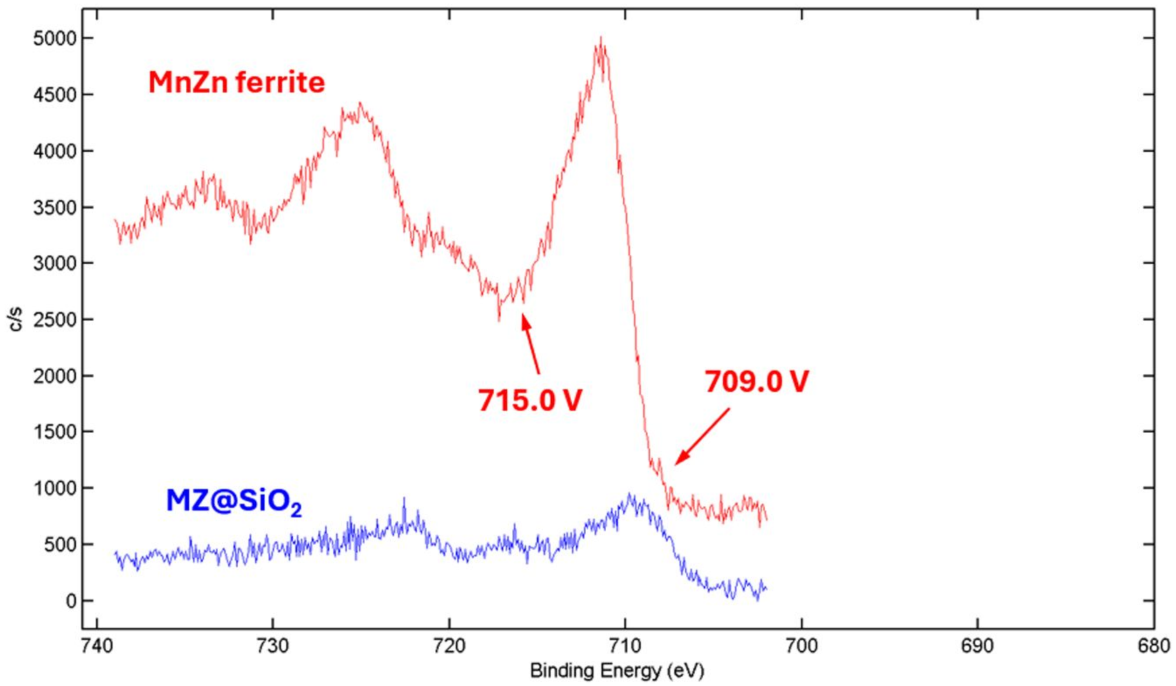

Figure S4. High-resolution Fe 2p XPS spectra of MnZn ferrite before and after SiO_2 coating (denoted as MZ@SiO_2).

The MnZn ferrite spectrum (red) shows two dominant peaks at ~711 eV (Fe 2p3/2) and ~725 eV (Fe 2p1/2), along with weak features at ~709 eV and ~715 eV corresponding to Fe^{2+} contributions and their associated satellite peaks. After SiO_2 coating, the Fe 2p signal intensity (blue) is significantly attenuated, confirming the formation of a conformal SiO_2 shell that suppresses surface Fe emission without altering the underlying oxidation state.

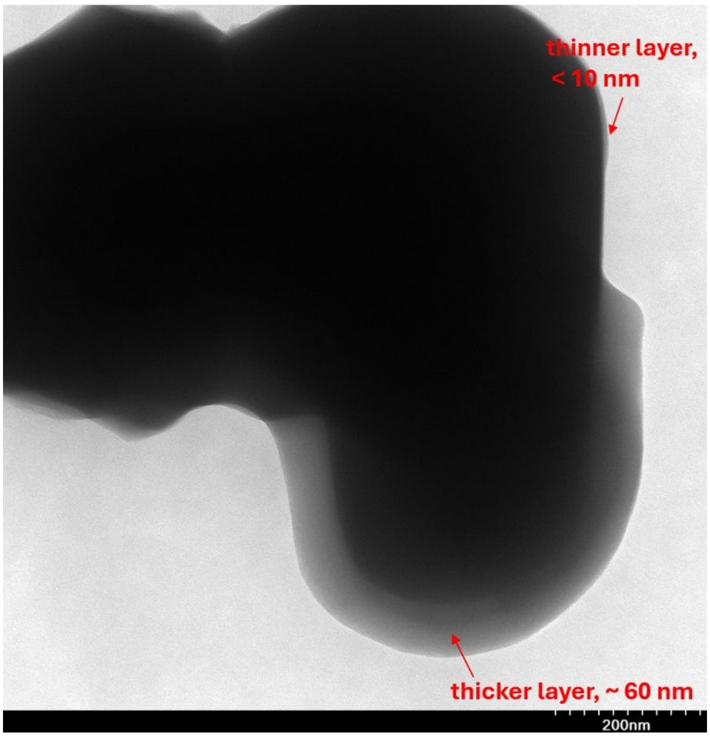

Figure S5. TEM image of the MnZn ferrite particles coated with SiO_2 (MZ@SiO_2).

As shown in Figure S5, the ferrite particle is encapsulated by a SiO_2 layer with a thickness ranging from below 10 nm in thinner regions to approximately 60 nm in thicker ones. This variation likely originates from the irregular morphology of the MnZn ferrite particles, where regions with higher surface curvature exhibit greater surface energy, promoting preferential adsorption of silane precursors during the sol-gel process. Consequently, localized hydrolysis and condensation reactions occur more extensively on these high-energy areas, forming thicker coatings, while smoother regions develop thinner layers. Such morphology-dependent coating behavior is commonly observed in Stöber-derived silica systems.

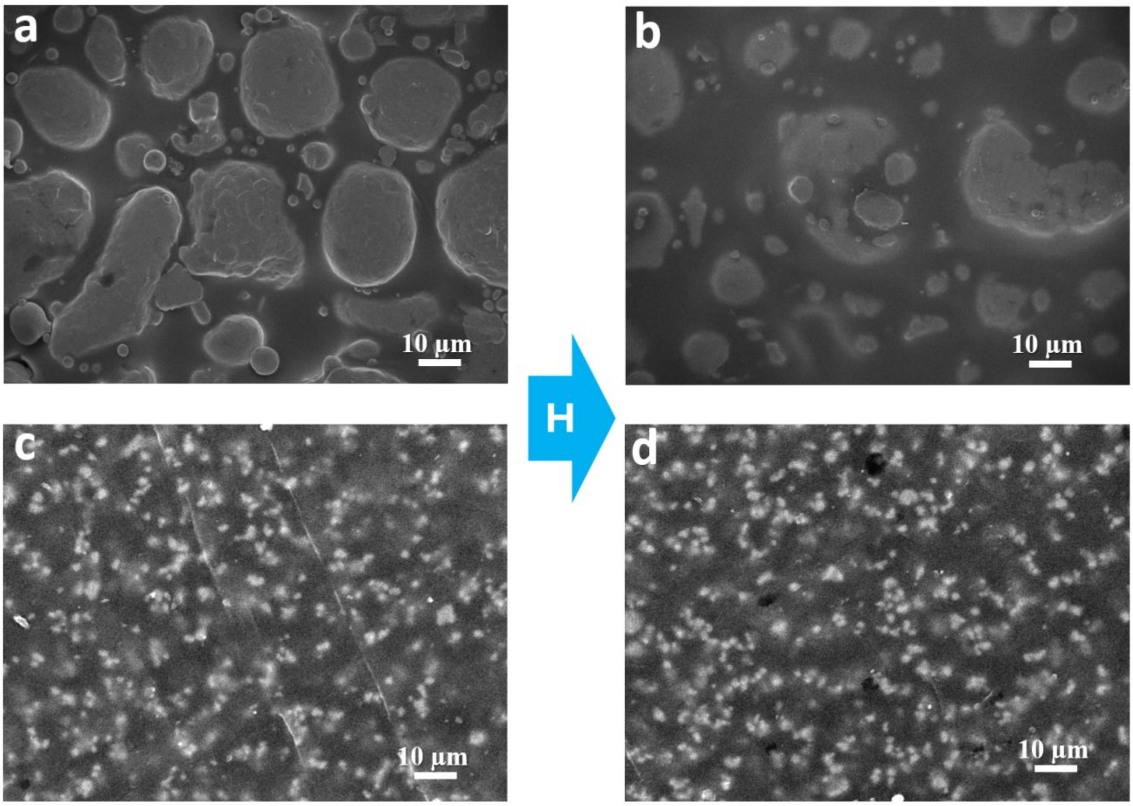

Figure S6. Cross-sectional SEM images of magnetic films: (a,b) MZ@SiO_2/GPTMS/epoxy and (c,d) hybrid of MZ@SiO_2 and FeNi alloy (3:1 v/v) in GPTMS/epoxy; (a,c) before and (b,d) after particle alignment by an external magnetic field of 0.04 T.

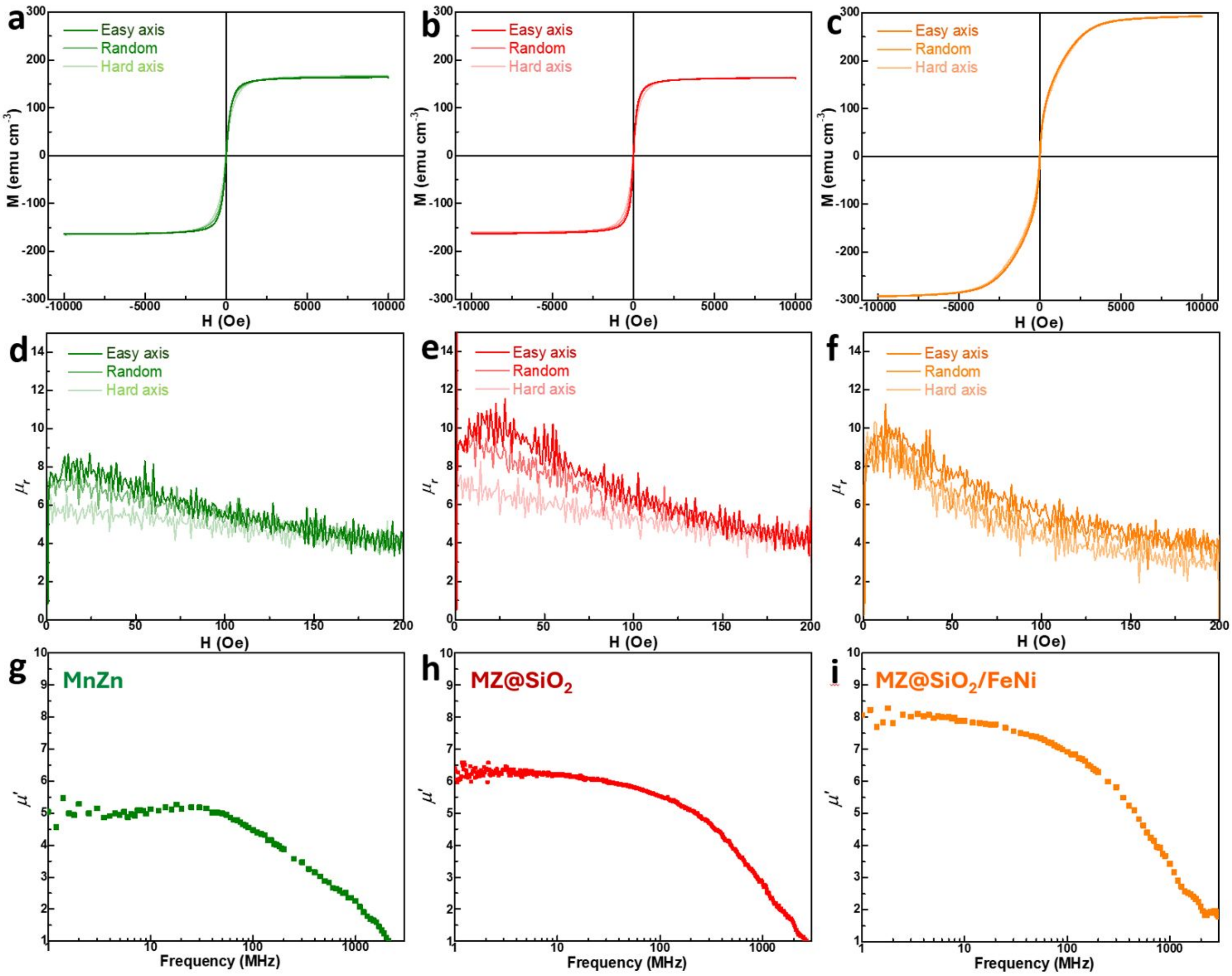

Figure S7. Magnetic and frequency-dependent properties of three types of composite films: (a,d,g) MnZn ferrite/GPTMS/epoxy, (b,e,h) MZ@SiO$_2$/GPTMS/epoxy, and (c,f,i) hybrid MZ@SiO$_2$/FeNi (3:1 v/v) in GPTMS/epoxy. (a-c) M–H hysteresis loops measured under different particle alignment orientations (easy, hard, and unaligned). (d-f) Relative permeability (μ_r) as a function of applied magnetic field strength, and (g-i) real component of permeability (μ') versus frequency, demonstrating the enhancement in magnetic response after SiO$_2$ coating and FeNi incorporation.

The μ' values shown in Figure S7(g,h,i) correspond to measurements taken along the easy axis of the three films.

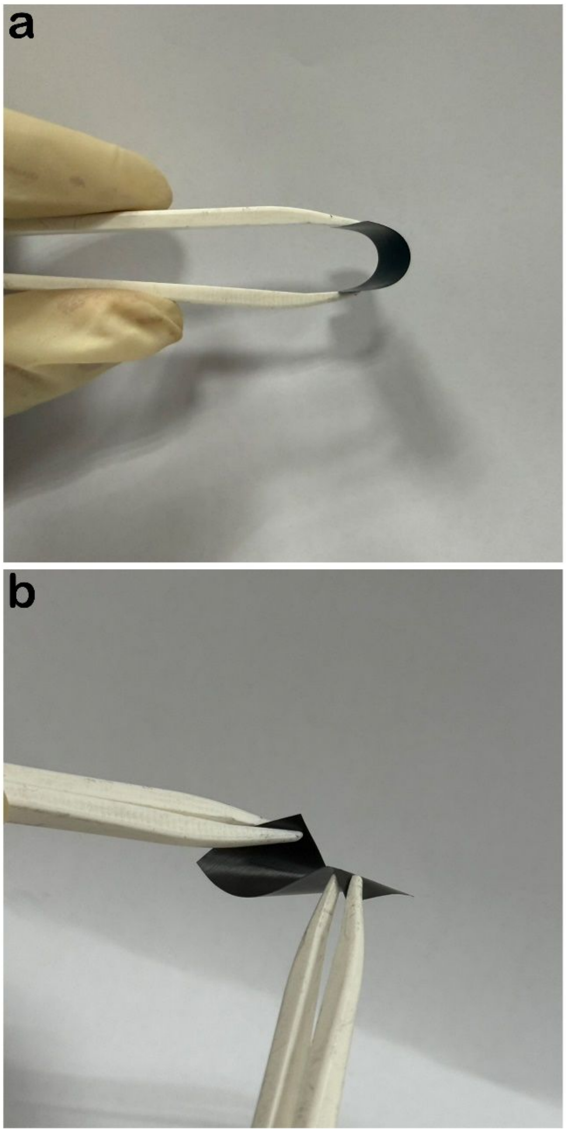

Figure S8. Photographs demonstrating the flexibility of the fabricated hybrid film composed of $MZ@SiO_2$ and FeNi alloy (3:1 v/v), showing its ability to (a) bend and (b) twist without visible cracking. The solid content of the magnetic powder in the composite film is 50 vol%.

The other two composite films, composed of MnZn ferrite and $MZ@SiO_2$, exhibit mechanical flexibility comparable to that of the hybrid $MZ@SiO_2$/FeNi alloy film shown in Figure S8 and are therefore not displayed here to avoid redundancy. The videos corresponding to the flexibility demonstrations in Figure S8(a) and S8(b) are provided as Videos S1 and S2, respectively.

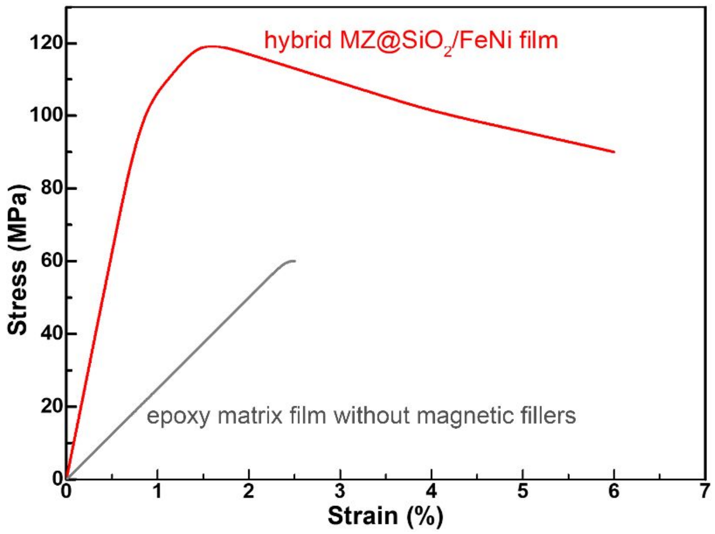

Figure S9. Stress-strain curves of the hybrid MZ@SiO_2/FeNi composite film and the bare epoxy film.

Figure S9 compares the tensile stress-strain curves of the hybrid MZ@SiO_2/FeNi composite film and the bare epoxy film. The hybrid composite exhibits a significantly higher ultimate tensile strength (~120 MPa) and greater strain at break (~6%) compared to the bare epoxy film (~60 MPa, ~2%), confirming its superior mechanical robustness and flexibility. This result aligns with the bending and twisting behaviors observed in Figure S8. The improved strength and ductility are expected to arise from the strong interfacial adhesion between the SiO_2-modified ferrite particles and the epoxy matrix, which promotes efficient load transfer and inhibits crack propagation under tension. Besides, using the conventional bending-strain relationship

$$\varepsilon = t/2R \qquad \text{(S1)}$$

where ε is the surface strain, t is the film thickness (~100 μm), and R is the bending radius, the measured strain-at-break of approximately 6% corresponds to a minimum bending radius of around 0.8 mm. This minimal radius demonstrates that the fabricated composite film possesses outstanding flexibility and mechanical resilience suitable for flexible electronic and magnetic device applications.
